# CCL5 deficiency aggravates acute DSS-induced colitis by restricting IL-33-induced formation of Tregs in intestinal tract

**DOI:** 10.1042/CS20256734

**Published:** 2026-01-07

**Authors:** Yang Luo, Ting-Yue Gong, Yong-Heng Zhao, Hao Li, Guang-Yao Ye, Zi-Zhen Zhang, Min-Hao Yu, Yan Zhang, Ming Zhong

**Affiliations:** 1 Department of Gastrointestinal Surgery, Ren Ji Hospital, School of Medicine, Shanghai Jiao Tong University, Shanghai, P.R., 200127, China; 2 State Key Laboratory of Oncogenes and Related Genes, Renji-Med X Clinical Stem Cell Research Center, Ren Ji Hospital, School of Medicine, Shanghai Jiao Tong University, Shanghai, P.R., China; 3 Department of General Surgery, Gongli Hospital, 219 Miaopu Road, Pudong New District, Shanghai, P.R., 200135, China

**Keywords:** CCL5, IL33, Tregs formation, acute DSS-induced colitis, ulcerative colitis

## Abstract

Ulcerative colitis (UC) is a chronic inflammatory disease of the gastrointestinal tract, characterized by ongoing intestinal inflammation, epithelial damage, and mucosal injury. Despite the identification of C-C motif chemokine ligand 5 (CCL5) as a key mediator in UC, the precise mechanisms underlying its role in immune activation and inflammation remain unclear. This study aimed to investigate CCL5 as a critical immune modulator in UC, focusing on its effects on immune cell activation, particularly regulatory T cell (Treg) formation, and the molecular pathways involved in these processes. Using the dextran sulfate sodium salt (DSS)-induced UC model and CCL5 knockout (*Ccl5*-KO) mice, we demonstrated that CCL5 deficiency exacerbates intestinal inflammation during the acute phase of colitis, partly due to impaired interleukin-33 (IL-33)-induced Treg formation. In addition, we observed a positive correlation between CCL5 expression and forkhead box protein 3 (FOXP3) levels in inflamed colon tissues of UC patients, suggesting a role for CCL5 in Treg regulation. Mechanistically, CCL5 deficiency disrupted the PI3K/Akt/NF-κB signaling pathway, resulting in reduced IL-33 expression, which in turn impaired CD4^+^ T cell activation and FOXP3^+^ Treg formation via the JAK1/STAT5 pathway. *In vivo* rescue experiments confirmed that restoring IL-33 signaling could alleviate inflammation and partially recover Treg function. Collectively, these findings highlight CCL5 as a novel immune modulator of Treg formation and immune responses in UC and suggest that targeting CCL5 may offer a promising therapeutic strategy for managing UC and related inflammatory diseases.

## Introduction

Ulcerative colitis (UC) is one of the main forms of inflammatory bowel disease (IBD) and is characterized by an idiopathic, chronic colonic mucosal inflammatory disorder, which starts in the rectum and generally extends proximally in a continuous manner through part of, or the entire, colon. Although the precise etiology of UC remains unclear, genetically susceptible individuals are more likely to suffer from aberrant intestinal inflammation [1]. CD4^+^ T cells, also known as helper T cells (Th), are a group of polymorphic and plastic T cells, including Th1, Th2, Th17, and regulatory T cells (Tregs), capable of responding to changes in the gut microenvironment and play a vital role in intestinal immune chain [2]. In the 1990s, it was well acknowledged that IBD is a typical Th1-related disease [3]. Nowadays, despite cumulative in-depth studies revealing the essential pathophysiological roles of Th17 and Tregs in modulating intestinal immune response during inflammation progression, the underlying mechanism remains unclear.

C-C chemokine motif ligand 5 (CCL5), also known as Regulated upon Activation, Normal T Cell Expressed and Presumably Secreted (RANTES), belonging to C-C subfamily of chemokines, functions as a professional chemoattractant that induces both *in vitro* and *in vivo* migration and recruitment of T cells, dendritic cells, eosinophils, NK cells, mast cells, and basophils into inflammatory lesions in various pathological processes [4]. CCL5 can be generated by platelets, macrophages, eosinophils, fibroblasts, endothelial, epithelial, and endometrial cells, despite originally being thought to be a T cell-specific cytokine [5]. The variety of cells that express and mediate CCL5 effects implicates the diversity of CCL5 functions in multiple biological processes, from pathogen control to enhancement of inflammation in several ‘sterile’ disorders, such as cancer [6] and atherosclerosis [7]. Nowadays, CCL5 has been studied in many diseases such as inflammation [8], cancers [9], virus infection [10], and immune responses [11]. It has been reported that IBDs may be related to the CCL5/CCR5 axis. The CCL5 level induced an influx of inflammatory factors, indicating the undying role of CCL5 in the pathogenesis of IBD [12]. However, the mechanism behind it remains to be explored.

IL-33, a member of the IL-1 cytokine family, plays a critical role in immune regulation by promoting the activation and maintenance of Tregs, especially in inflammatory environments. It signals through its receptor ST2, which is expressed on various immune cells, including Tregs [13]. IL-33 binding to ST2 enhances Foxp3 expression, crucial for Treg differentiation and function, and promotes Treg survival and expansion in inflammatory conditions such as colitis and autoimmune diseases [14,15]. Additionally, IL-33 synergizes with other cytokines like TGF-β to bolster Treg activity, thereby helping to control effector T cell responses and tissue inflammation [16]. Despite these findings, the exact mechanisms through which IL-33 influences Tregs in acute diseases remain unclear, particularly regarding its interaction with other inflammatory factors like CCL5. CCL5 has been shown to recruit immune cells during inflammation, but its interaction with IL-33 and its potential role in modulating Treg responses has not been fully explored. Understanding how these two molecules interact could provide insights into novel therapeutic approaches for managing acute inflammatory diseases, especially by enhancing immune tolerance.

Our previous study has demonstrated that in luminal breast cancer, CCL5 could induce the Th2 polarization of CD4^+^ T cells to promote pulmonary metastasis [17]. Here, the aim of this study was to investigate the role of CCL5 in the regulation of inflammatory responses in UC. Specifically, we sought to understand how CCL5 influences immune cell activation, particularly Treg formation, and the molecular pathways involved in these processes. Using a DSS-induced UC mouse model, we found that genetic knockout of CCL5 exacerbates intestinal inflammation by disturbing IL-33 levels and impairing subsequent FOXP3^+^ Treg formation. In addition, analysis of clinical samples from UC patients confirmed a significant increase in CCL5 expression, which correlated positively with FOXP3 expression, suggesting a role for CCL5 in Treg regulation. Mechanistically, CCL5 deficiency disrupted the PI3K/Akt/NF-κB signaling pathway, resulting in reduced IL-33 expression. This, in turn, impaired CD4^+^ T cell function and decreased FOXP3^+^ Treg formation via the JAK1/STAT5 signaling pathway. Finally, through *in vivo* rescue experiments, we demonstrated that restoring IL-33 signaling could alleviate inflammation and partially recover Treg function, further confirming the protective role of CCL5 during acute colitis. These findings contributed to the understanding of the immune mechanisms underlying UC and provided a theoretical basis for the development of novel therapeutic strategies.

## Materials and methods

### Patients and tissues

UC samples were obtained from patients at Renji Hospital (Shanghai, China). Thirty-two patients with UC were studied, and the mean age was 33.84 ± 2.09 years (range: 16–65 years) (**Supplementary Table S1**). All human research was carried out in accordance with the World Medical Association Declaration of Helsinki, and written informed consent was obtained from all participants prior to their participation in the study. All experiments were approved by the local ethics committee of the Shanghai Jiao-Tong University School of Medicine at Renji Hospital (Approval number: LY2023-016-B).

### Mice and induction of intestinal inflammation

**Figure 1:**
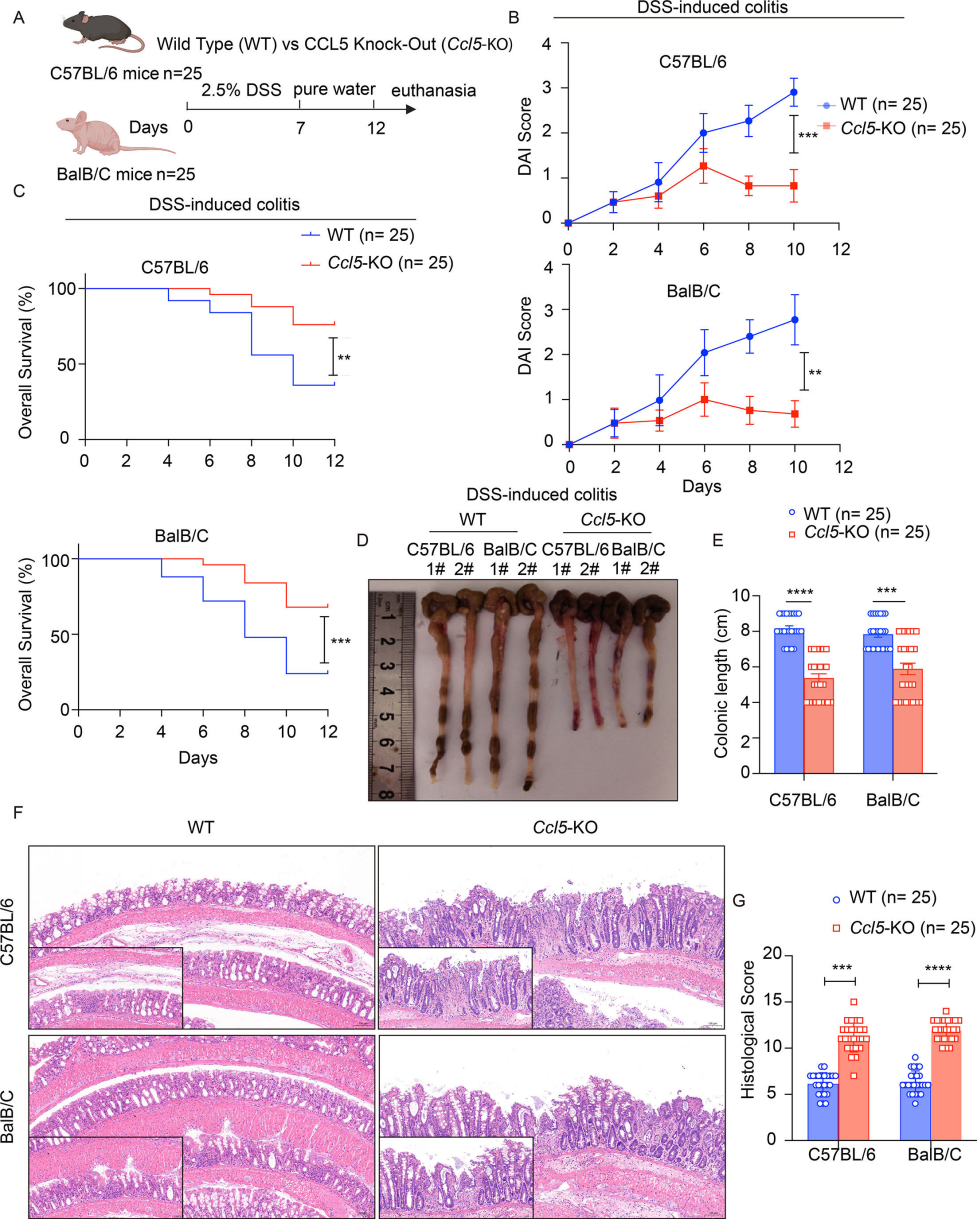
C-C motif chemokine ligand 5 (CCL5) deficiency exacerbates dextran sulfate sodium salt (DSS)-induced colitis. Wildtype (WT) and CCL5 knockout (*Ccl5*-KO) mice of both C57BL/6 and BalB/C backgrounds were subjected to 7-day 2.5% DSS administration in the drinking water. (**A**) Schematic of construction of DSS-induced colitis model. (**B**) Disease activity index (DAI), *n* = 25 per group. (**C**) Survival rate of WT (*n* = 25) and *Ccl5*-KO (*n* = 25) mice was observed for 2 weeks, and the corresponding survival curves were plotted. (**D and E**) Gross anatomy of colons (**D**) and colon length (**E**) were monitored. Colon length was measured in both the C57BL/6 and BalB/C mouse strains after 7 days of 2.5% DSS treatment. The results are shown as the mean ± SEM, with statistical significance assessed using one-way analysis of variance (ANOVA) followed by Tukey’s post hoc test. (**F and G**) Representative H&E staining (**F**) of colon sections from WT and *Ccl5*-KO mice 4 days after 7-day 2.5% DSS administration (scale bars: 50 μm, 100 μm). Histological score (**G**) was quantified. Results shown are the mean ± SEM (ns, nonsignificant; **P<0.01, ***P<0.001, ****P<0.0001) of triplicate determination from three independent experiments.

CCL5 knockout (*Ccl5*-KO) mice and wildtype (WT) mice were obtained from The Jackson Laboratory. In the initial phenotypic experiments (Figure 1), 25 mice in each group aged 6–8 weeks, including 12 females and 13 males, were used to ensure adequate statistical power and to account for sex-based variability. Both BalB/C and C57BL/6 mice were treated with 2.5% dextran sulfate sodium salt (DSS, molecular mass = 40,000 Da; MP Biomedicals, USA, Cat#: MFCD00081551; CAS: 9011-18-1) in sterile water as the only source of drinking water for 7 days. This treatment induced reproducible colitis, characterized by reduced body weight, rectal bleeding, mucosal ulceration, crypt destruction, and leukocyte infiltration. Control mice received pure drinking water, and all mice were weighed and monitored daily for signs of sickness. The disease activity index (DAI, 0–4) was determined by assessing weight loss compared with initial weight, stool consistency, and rectal bleeding. The DAI scoring system was as follows: weight loss: 0 (no loss), 1 (1–5%), 2 (5–10%), 3 (10–20%), and 4 (>20%); stool consistency: 0 (normal), 2 (loose stool), and 4 (diarrhea); and bleeding: 0 (no blood), 2 (visual pellet bleeding), and 4 (gross bleeding, blood around anus). At the end of the experimental period, mice were anesthetized with CO_2_, killed, and their intestinal tissues were collected for subsequent analysis. All animal experiments were conducted in compliance with the regulations of the Association for the Assessment and Accreditation of Laboratory Animal Care in Shanghai and were carried out at the East China Normal University Center for Animal Research (Approval number: m20241002).

### *In vivo* treatments

To rescue the phenotypes of enteritis, exogenous recombinant murine CCL5 protein (HY-P71890, MCE) [18] was intraperitoneally injected at a concentration of 350 ng/kg from the onset of DSS treatment. To explore the regulation of IL-33 levels through the PI3K/Akt/NF-κB signaling pathway, *Ccl5*-KO mice in the DSS model were treated with nuclear factor kappa B (NF-κB) (P65) inhibitors, BAY 11-7082 (Cat#: B5556, Sigma-Aldrich, USA; CAS: 19542-67-7), and Akt inhibitor, Capivasertib (AZD5363) (Cat#: HY-15431, MCE, China; CAS: 1143532-39-1). For NF-κB inhibition, BAY 11-7082 was intraperitoneally administered twice a week at a dosage of 5 mg/kg during DSS treatment. For Akt inhibition, Capivasertib (AZD5363) was added to the drinking water at a concentration of 20 mg/kg. After 12 days of *in vivo* drug treatment, mice were killed, and their colons were collected for analysis. For *in vivo* treatments, each experimental group also contained six male mice, aged 6–8 weeks. During this period, the body weight and DAI of the mice were recorded. To assess the role of IL-33 [19], recombinant IL-33 (10 ng/µl, Soluble, Mouse, Recombinant, HY-P7218, MCE) was intraperitoneally injected daily at a dose of 100 µL (10 ng/µL) following 7 days of DSS treatment. rIL-33 was administered intraperitoneally at the aforementioned dosage daily from the onset of DSS induction. The mice were anesthetized using CO_2_, killed, and their intestinal tissues were harvested for further experimental analysis.

### Bone marrow chimera models

Bone marrow chimera models were constructed as reported [20]. In brief, bone marrow chimeras were constructed by transplanting bone marrow from the femurs and tibiae of WT and *Ccl5*-KO donor mice. Each group consisted of six male mice, aged 6–8 weeks. Recipient WT or *Ccl5*-KO mice were irradiated with 9 Gy of X-rays to remove the host hematopoietic system and reconstituted with 5 × 10^6^ bone marrow cells. Two months later, mice were bled to confirm chimerism. Four chimera groups were generated: WT recipient mice (WT^WT^ or WT^KO^) and KO recipient mice (KO^WT^ or KO^KO^). The mice were subjected to CO_2_ anesthesia, then killed, and their intestinal tissues were collected for subsequent analysis.

### Colon explant cultures

The colon was cut into 1 mm^3^ sections and then washed in PBS. The sections were placed in complete RPMI 1640 medium (Gibco, USA, Cat#: C11875500BT, with 10% fetal bovine serum [FBS] [Gibco, Cat#: 10270106], 1% penicillin and streptomycin [Gibco, Cat#: 15140122]) and cultured at 37°C for 8 hours. The supernatants were harvested, and cytokine levels in the supernatants were determined by enzyme-linked immunosorbent assay (ELISA) IL-33 (eBioscience, USA, Cat#: BMS6025TEN). Concentrations were normalized to the weight of the explants.

### Histological, immunohistochemical, and immunoﬂuorescent staining

The human and mice colon tissues were fixed in 10% buffered formalin (pH 7.4) for 24 hours at room temperature before processing for immunohistochemical (IHC) analysis. The tissues were then embedded in paraffin and sectioned at a thickness of 5 μm. A Vectastain Elite ABC Kit obtained from Vector Laboratories (USA, Cat#: PK-4001) was used for IHC staining according to the protocol recommended by the manufacturer. Mice colon tissues were embedded in paraffin for examination. Sections were stained with hematoxylin (Thermo Fisher Scientific, USA, Cat#: 51275-100ML) and eosin (Sigma, Germany, Cat#: P4707) (H&E), and histopathological scoring was performed based on the supplementary score table (**Supplementary Table S2**). For each category, the recorded score reflects the most severe finding. Briefly, all specimens were deparaffinized and rehydrated. To expose antigens, samples were unmasked by submerging them into boiling sodium citrate buffer (10 mmol/L, pH 6.0) for 10 minutes and then treated with 3% H_2_O_2_ for 10 minutes. The slide was blocked with 10% goat serum albumin in 1 × PBS in a humidified chamber for 1 hour at room temperature. The slides of human tissue array sections with FOXP3 (1:100) (Abcam, UK, Cat#: ab215206, RRID: AB_2860568) or CCL5 (1:100) (Cell Signaling Technology, USA, Cat#: 36467S), and the mouse colon tissue sections were hybridized with FOXP3 (1:100) or CD4 (1:100) (Abcam Cat#: ab183685, RRID: AB_2686917) at 4°C in a humidified chamber overnight. The slides were washed and hybridized with the secondary antibodies from Vector Laboratories (anti-rabbit 1:150, Cat#: BA-1000; anti-mouse 1:150, Cat#: BA-2000 or anti-rat 1:150, Cat#: BA-4000) for 1 hour at room temperature. Slides were stained using the Vectastain Elite ABC Kit (Vector Laboratories, Inc.). After developing with 3,3-diaminobenzidine, the sections were counterstained with hematoxylin. The tissue sections were observed under a Leica DM6 B bright-field microscope and analyzed using Image-Pro PLUS (v.6) computer software program (Media Cybernetics, Inc.).

For immunostaining, sections were boiled in 10 mM sodium citrate (pH 6.0) for 10–15 minutes for antigen retrieval before being stained with primary antibodies overnight. The primary antibody was incubated overnight, slides were washed and hybridized with the secondary antibodies for 1 hour. Primary antibodies (anti-NF-κB P65 antibody [Abcam, Cat#: ab32536, RRID: AB_776751]) were used. Secondary antibodies were goat anti-rabbit IgG, Alexa 594 (1:400 [Thermo Fisher Scientific, Cat#: A-11037, RRID: AB_2534095]), and DAPI (1:1000, Thermo Fisher Scientific, Cat#: D3571, CAS: 28718-90-3). Slides were then washed three times and, after the final wash, mounted onto slides in Vectashield (Vector Biolabs) for imaging using a Leica TCS SP8 confocal microscope. All washes were with PBS + 0.1% Triton X except for the final wash in PBS.

### Western blot

Western blot (WB) analysis was conducted following the procedure outlined in a previous publication [21]. Prior to performing the WB analysis, tissue samples were homogenized using a buffer containing protease inhibitors, followed by centrifugation at 12,000×g for 15 minutes at 4°C to remove cellular debris. The supernatant was collected for protein quantification. Proteins were separated by SDS-PAGE under reducing conditions and then transferred onto PVDF membranes using a wet transfer system at 100 V for 1.5 hours. Membranes were blocked with 5% nonfat dry milk in TBS containing 0.1% Tween-20 (TBST) for 1 hour at room temperature before incubation with primary antibodies overnight at 4°C. The following primary antibodies were utilized for the detection of target proteins: CCL5 (1:1000, Cell Signaling Technology Cat#: 36467, RRID:AB_3644243); FOXP3 (1:1000, Abcam, Cat#: ab215206, RRID: AB_2860568); IL33 (1:1000, Abcam, Mouse: Cat#: ab187060, RRID: AB_2894704/Human: Cat#: ab207737, RRID: AB_2827630); NF-κB p65 (1:1000, Abcam, Cat#: ab32536, RRID: AB_776751); Phospho-NF-κB p65 (Ser536) Monoclonal Antibody (1:1000, Thermo Fisher Scientific, Cat#: MA5-15160, RRID:AB_10983078); ST2 (1:1000, Invitrogen, Cat#: PA5-20077, RRID: AB_11156630); IkB alpha Monoclonal Antibody (1:1000, Thermo Fisher Scientific Cat#: MA5-15132, RRID:AB_10982641); Phospho-IkB alpha (Ser32, Ser36) Monoclonal Antibody (1:1000, Thermo Fisher Scientific Cat#: MA5-15224, RRID:AB_10981266); PI3K p85 alpha Monoclonal Antibody (1:1000, Thermo Fisher Scientific Cat#: MA1-74183, RRID:AB_2163452); Phospho-PI3K p85/p55 (Tyr458, Tyr199) Polyclonal Antibody (1:1000, Thermo Fisher Scientific Cat#: PA5-17387, RRID:AB_10985894); AKT Pan Polyclonal Antibody (1:1000, Thermo Fisher Scientific Cat#: 44-609G, RRID:AB_2533692); Phospho-AKT1 (Ser473) Monoclonal Antibody (1:1000, Thermo Fisher Scientific Cat#: 44-621G, RRID:AB_2533699); JAK1 Recombinant Rabbit Monoclonal Antibody (1:1000, Thermo Fisher Scientific Cat#: MA5-32780, RRID:AB_2810057); Phospho-JAK1 (Tyr1022, Tyr1023) Polyclonal Antibody (1:1000, Thermo Fisher Scientific Cat#: 44-422G, RRID:AB_2533648); STAT5A/B Polyclonal antibody (1:1000, Proteintech Cat#: 12071-1-AP, RRID:AB_2196933); Phospho-Stat5 (Tyr694) Antibody (1:1000, Cell Signaling Technology Cat#: 9351, RRID:AB_2315225); β-actin (1:10,000; Abcam, Cat#: ab6276, RRID: AB_2223210).

### Isolation of immune cells from mouse lymphoid tissue and blood

Single-cell suspensions of lymphocytes from mouse spleen and peripheral blood and intestinal lamina propria lymphocytes (LPLs) were obtained as previously reported [22,23]. Briefly, splenic cells were obtained by gentle pressure dissociation of spleen using fluorescence-activated cell sorting (FACS) buffer (0.1% bovine serum albumin [BSA], 2 mM ethylenediaminetetraacetic acid [EDTA], and 0.02% azide adjusted with D-PBS) and then passed through a 100 mm sterile cell strainer. Red blood cells were lysed by addition of 5 mL RBC lysis buffer (BioLegend, San Diego, CA, USA), mixed briefly to resuspend cells, and incubated for 5  minutes at room temperature, followed by FACS buffer wash and spin. LPLs were isolated from the colon. Initially, colons were cleansed with PBS for fecal removal, longitudinally opened, and sectioned into 1 cm fragments. These fragments underwent a dual wash in PBS with 3 mmol/L EDTA at 37°C for 10 minutes, followed by two additional rinses in RPMI 1640 (containing 1% FBS, 1 mM ethylene glycol tetraacetic acid [EGTA], and 1.5 mmol/l MgCl_2_) for 15 minutes at 37°C to eliminate EDTA. The colon sections were then washed in PBS, minced, and enzymatically decomposed in RPMI 1640 with 20% FBS and 100 U/mL collagenase Type IV (Merck, Cat#: C4-28-100MG) for 60 minutes at 37°C. Post-digestion, the mix was filtered through a 40 µm mesh to segregate single cells from residual tissues. The isolated cells were then cleansed in RPMI 1640 and suspended in culture medium for subsequent analysis.

For preparation of lymphocytes from lymph nodes or spleen for negative selection, freshly collected spleen or lymph nodes were placed in a 70 μm cell strainer pre-wetted with PBS on top of 50 mL centrifuge tube. The thumb side of a 10 mL syringe plunger was then used to smash the spleen and lymph nodes, while constantly adding up to 5 mL PBS. The isolated cells were washed twice with PBS and resuspended in negative selection buffer at a density of 10^8^ cell/mL.

### Isolation of gut-associated lymphoid tissues from UC surgical tissue specimens

For obtaining immune cells from surgical tissue specimens removed from UC patients, intestinal surgical tissues were cut into 5–7 cm^2^ pieces, and removed remaining mucus and fecal material by incubating each tissue piece in 10 mL warm R5 medium (add 50 mL of FBS and 1 × Antibiotic–Antimycotic to 445 mL of RPMI 1640) with 4 mM 1,4-Dithiothreitol (DTT, Sigma-Aldrich, Cat#: D8255; CAS: 6892-68-8) (DTT medium: Warm R5 medium to 37°C and add 4 µL DTT per mL R5 medium) at 37°C under constant agitation (350 rpm) on an orbital shaker for 10 minutes. Identification and isolation of submucosal and LP-embedded mucosal isolated lymphoid follicles and Peyer’s patch follicles were referred to guidelines as reported [24]. Obtained follicles were incubated in 200–500 µL R5 medium with 0.154 mg/mL DNAse I and 0.062 mg/mL Liberase TM (Sigma, Cat#: 05401127001) at 37°C with constant agitation (350 rpm) on a heating block for 45 min. The resulting material was washed with at least 5 mL of ice-cold R5 medium through a 100 µm filter. The isolated cells were spinned down at 400 ***g*** for 5–7 min at 4°C and resuspended pellet(s) in 1 mL R5 medium for further analysis.

### Isolation of human intestinal epithelial and stromal cells

Human colonic epithelial and stromal cells were isolated according to previously established protocols [25], as briefly described here: Tissue specimens were subjected to multiple washes in calcium- and magnesium-free Hanks’ Balanced Salt Solution (CMF-HBSS; Gibco, Cat#: 14175095). The mucosal layer was meticulously separated from the submucosal layer and subsequently washed for 30 minutes in 0.04% sodium hypochlorite solution. Following separation, mucosal strips were sectioned into 1 cm² pieces and incubated for 1.5 hours at 22°C in a solution containing 1 mmol/L EDTA (Gibco, Cat#: AM9912), 1 mmol/L EGTA (MCE, Cat#: HY-D0861), and 0.5 mmol/L dithiothreitol (MCE, Cat#: HY-15917) in PBS. Epithelial cells, preserved as intact crypts, were detached by administering 10 vigorous agitations of the container. This detachment procedure was replicated six times, each with 10 mL aliquots of CMF-HBSS. Liberated crypts were collected by centrifugation, washed once in PBS, and the residual tissue was returned to the original Eppendorf tube, identified as stromal (S). Stromal cells were isolated using an enzymatic mixture (HBSS [Gibco, Cat#: 14175-079]; Liberase-TH [0.13 WU/mL, Sigma, Cat#: 5401135001]; DNase I [0.5 U/mL, Roche, Cat#: 10104159001]) applied across three digestion cycles. Contents were transferred from the collection tube to a new 15 mL conical tube and centrifuged at 700 ***g*** for 3 minutes. Subsequently, stromal cells were collected by resuspending the pellet in 0.5 mL of thaw medium (DMEM [Corning, Cat#: 10-013-CV] supplemented with 10 mM HEPES [Gibco, Cat#: 15630-080], 5% FBS [Gibco, Cat#: 10270106], and 1 × Antibiotic–Antimycotic [Gibco, Cat#: 15240-062]) and filtered into FBS-primed tubes. Epithelial cells were collected by centrifuging the crypt suspension at 700 × g for 1 minute and reconstituting the pellet in 1.5 mL of TrypLE (Gibco, Cat#: 12605-010) supplemented with 15 μL of DNase I. The mixture was then incubated in a ThermoMixer for 30 minutes at 37°C and 800 rpm. Finally, epithelial cells were washed in thaw medium and resuspended in 500 μL of HBSS supplemented with 1% BSA (Fisher Bioreagents, Cat#: BP1600-100) and 5 μL of DNase I.

### Flow cytometric analysis

For surface staining, the viable cells selected by Fixable Viability Dye eFluor 506 (eBioscience, Cat# 65-0866-14) were incubated with the following fluorochrome-conjugated monoclonal antibodies: anti-mouse CD45 (Clone ID 30-F11) APC-Cy7 (BD Biosciences, Cat#: 557659; RRID: AB_396774), anti-mouse CD4 APC-eFluor 780 (RM4-5) (eBioscience, Cat#: 47-0042-82; RRID: AB_1272183), anti-mouse CD8a PE-Cy7 (53-6.7) (eBioscience, Cat#: 25-0081-82; RRID: AB_469584), anti-mouse CD11b Monoclonal Antibody (M1/70), PE (eBioscience, Cat#: 12-0112-82, RRID:AB_2734869); Anti-human CD45-FITC (HI30) (eBioscience, Cat#: 11-0459; RRID: AB_10852703), anti-human CD4 Monoclonal Antibody (RPA-T4), FITC (eBioscience, Cat#: 11-0049-42, RRID:AB_1659694), anti-human CD8 Monoclonal Antibody, APC (BioLegend, Cat#: 344721, RRID:AB_2075390) for 20 minutes at 4°C. For intracellular FOXP3 expression, cell surface markers were stained as described above, and intracellular staining by Foxp3/Transcription Factor Staining Buffer Kit (Thermo Fisher Scientific, Cat#: 00-5523-00). For detection, cells were incubated with the anti-mouse FoxP3 APC (FJK-16s) (eBioscience, Cat#: 17-5773-82; RRID: AB_469456) and anti-human CD25 Antibody, PE (BioLegend, Cat#: 356147, RRID: AB_2890783) overnight at 4°C. Cells were analyzed using a Fortessa flow cytometer (BD Biosciences) and FlowJo software (Tree Star, Ashland, OR, USA).

### Negative selection of CD4^+^ T cells

CD4^+^ T cells were negatively selected from intestinal lymph nodes utilizing the Mouse CD4^+^ T Cell Isolation Kit (480033, BioLegend, San Diego, CA, USA). The lymphocytes, isolated as described above, were first adjusted to ≤10^8^ cells/mL in negative selection buffer. A biotin-antibody cocktail, composed of biotin-labeled anti-CD8a, CD11b, CD11c, CD19, CD24, CD45R/B220, CD49b, CD105, I-A/I-E (MHC II), TER-119/Erythroid, and TCR-γδ, was diluted at a 1:10 ratio into the cell suspension. After a 15-minute incubation on ice, an equal volume of freshly vortexed streptavidin magnetic nanobeads was introduced into the cell suspension, followed by another 15-minute incubation on ice. The cell suspension was subsequently transferred to 12 × 75 mm round-bottom polystyrene tubes, where the total volume was increased to 2.5 mL with negative selection buffer. The tube was placed in the magnet (MAG-4902-10, Thermo Fisher Scientific, Waltham, MA, USA) for 5 minutes for magnetic separation, before the collection of fluid containing unbound cells. This process was repeated using an additional 2.5 mL of negative selection buffer. The unbound fractions, enriched in CD4^+^ T cells, were pooled.

### RNA isolation and quantitative real-time polymerase chain reaction

RNA was isolated from *ex vivo* surgically removed intestinal tissues, using TRIzol (Thermo Fisher Scientific) according to the manufacturer’s instructions. cDNA was synthesized from 1 mg of RNA using random primers and the SuperScript III First-Strand Synthesis System (Invitrogen). Gene expression analysis was performed by means of quantitative real-time polymerase chain reaction using iTaq Universal SYBR Green Supermix (Bio-Rad, Cat#: 1725121), and all expression values were normalized to β-actin RNA levels. Primer sequences used：

*Ccl5*: 5’-CCTGCTGCTTTGCCTACATTGC-3’(Forward) and 5’ -ACACACTTGGCGGTTCTTTCGG-3’ (Reverse); *Foxp3*: 5’-GGCACAATGTCTCCTCCAGAGA-3’ (Forward) and 5’- CAGATGAAGCCTTGGTCAGTGC-3’ (Reverse).

### Statistical analysis

Data are presented as means ± SEM (standard error of the mean). All experiments were conducted with at least two independent biological replicates. All bioinformatic analyses were performed using biological triplicates. Statistical analyses were conducted using GraphPad Prism v9.1.0. To ensure the appropriateness of the unpaired two-tailed Student’s t-test, normality was assessed using the Shapiro–Wilk test, and homogeneity of variance was evaluated with Levene’s test. If the data met the assumptions of normality and homogeneity of variance, the unpaired two-tailed Student’s t-test was applied for two-group comparisons. If the data did not meet these assumptions, non-parametric tests (Mann–Whitney U test) were employed. For comparisons involving three or more groups, one-way or two-way analysis of variance (ANOVA) was used. If ANOVA indicated significant differences, post hoc analysis was performed using Tukey’s multiple comparison test to identify specific group differences. Pearson’s correlation coefficient was calculated to evaluate the linear relationship between CCL5 and FOXP3 expression. Prior to conducting Pearson’s correlation, linearity was confirmed using scatter plots and linear regression analysis. If the data did not meet the assumptions of linearity or normality, Spearman’s rank correlation was used as an alternative. Survival rates of animals were estimated using the Kaplan–Meier method, and group differences were assessed using the log-rank test. A *P*-value of <0.05 was considered statistically significant.

## Results

### CCL5 deficiency exacerbates DSS-induced colitis

To evaluate the effects of CCL5 on colitis development, genetic deletion of CCL5 in mice of both BalB/C and C57BL/6 backgrounds was constructed. Subsequently, we used an established murine model of colitis (UC mouse model) induced by oral administration of DSS, a reagent that disrupts the barrier function of mucosal epithelial cells, which leads to massive invasion and leakage of intestinal microorganisms and secretions into intestinal submucosal layer, in turn leading to intestinal damage and inflammation with manifestations of UC-like symptoms [26] (Figure 1A). Mice receiving pure water without DSS treatment (WT and *Ccl5*-KO, both BalB/C and C57BL/6 backgrounds) served as negative controls and did not exhibit any signs of inflammation or tissue damage, confirming that the observed pathological changes were DSS-specific. The results showed that after DSS administration, single-housed *Ccl5*-KO mice developed more severe colitis than WT mice on both genetic backgrounds, as indicated by increased DAI, including severe diarrhea, intestinal bleeding, and weight loss (Figure 1B). The log-rank test revealed reduced overall survival in *Ccl5*-KO mice (Figure 1C). *Ccl5*-KO mice exhibited significant intestinal shortening (Figure 1D and E), accompanied by greater inflammatory infiltration and crypt damage as shown by H&E staining (Figure 1F and G). Taken together, these data indicate that CCL5 deficiency exacerbates DSS-induced colitis, suggesting that CCL5 plays an important role in maintaining intestinal homeostasis during colitis.

### CCL5 plays a major role in modulating DSS-induced inflammation in both immune and nonimmune cells

**Figure 2:**
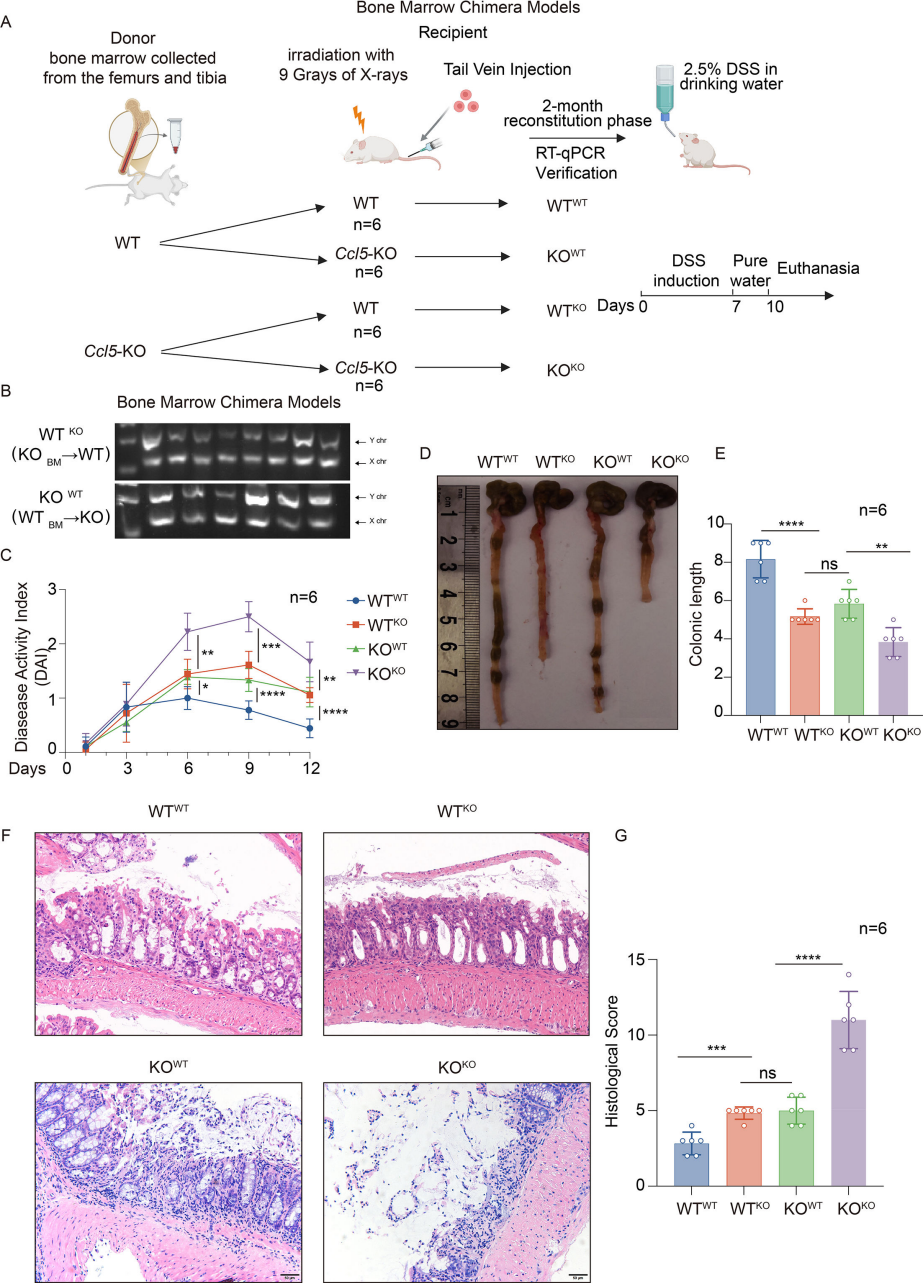
C-C motif chemokine ligand 5 (CCL5) plays a major role in modulating dextran sulfate sodium salt (DSS)-induced inflammation in both immune and nonimmune cells. (**A**) Schematic of construction of bone marrow chimera model. (**B**) Quantitative real-time polymerase chain reaction (RT-qPCR) verification for construction of bone marrow chimera mouse model. (**C**) Disease activity index (DAI) was recorded after a 2-month reconstitution phase, and reconstructed mice were administered with DSS for comparative analyses of colitis phenotypes, *n* = 6 per group. (**D and E**) Representative photo of colon (**D**) and colon length measurements (**E**) on day 10, *n* = 6 per group. (**F and G**) Representative H&E staining (**F**) of colon tissue sections from reconstructed mice (WT^WT^, WT^KO^, KO^WT^, KO^KO^) 4 days after 7-day 2.5% DSS administration (scale bars, 50 μm). Histological score (**G**) was quantified. Results shown are the mean ± SEM (ns, nonsignificant; *P<0.05, **P<0.01, ***P<0.001, ****P<0.0001) of triplicate determination from three independent experiments.

To investigate the cellular origins of changing CCL5 in the intestine of the UC mouse model, we initiated a bone marrow chimera mouse model [20,27]. Bone marrow cells collected from WT or *Ccl5*-KO mice were transferred into lethally irradiated WT or *Ccl5*-KO recipient mice via injection into the tail vein (Figure 2A). Real-time polymerase chain reaction (RT-PCR) results validated the successful construction of the transplantation model (Figure 2B). After a 2-month reconstitution phase, recipient mice were administered DSS for comparative analyses of colitis phenotypes. Through the close record of DAI, we found that in WT mice that received *Ccl5*-KO mouse bone marrow (WT^KO^) and *Ccl5*-KO mice that received WT mouse bone marrow (KO^WT^), the presence of CCL5 from transplanted nonimmune intestinal cells or from the host itself mitigated the severity of DSS-induced colitis. Meanwhile, the severity of colitis was highest in *Ccl5*-KO mice that received *Ccl5*-KO bone marrow (KO^KO^), lacking CCL5 from both sources: corresponding DAI was significantly higher than in the other three groups, with substantial weight loss and pronounced symptoms of diarrhea and fecal bleeding (Figure 2C). Gross pathology showed that the intestines were notably shortened in the KO^KO^ group (Figure 2D and E). Histological analysis further supported our findings that the WT^KO^ and KO^WT^ groups exhibited markedly less intestinal inflammation compared with the KO^KO^ group, which was more susceptible to DSS-induced colitis. The latter group exhibited severe tissue damage, characterized by a complete loss of crypt architecture in large, continuous areas and severe transmural extensions of inflammatory cell infiltrations (Figure 2F and G). It is noteworthy that in the WT^KO^ and KO^WT^ groups, the colitis phenotype was not completely reversed, suggesting that CCL5 from both immune and nonimmune cells plays a crucial role in modulating the severity of DSS-induced colitis. This finding underscores the complex interplay between different cellular environments and the immune signaling mediated by CCL5.

### CCL5 deficiency impairs CD4^+^ T cell activity by reducing the number of FOXP3^+^ Tregs in the inflamed intestine

**Figure 3:**
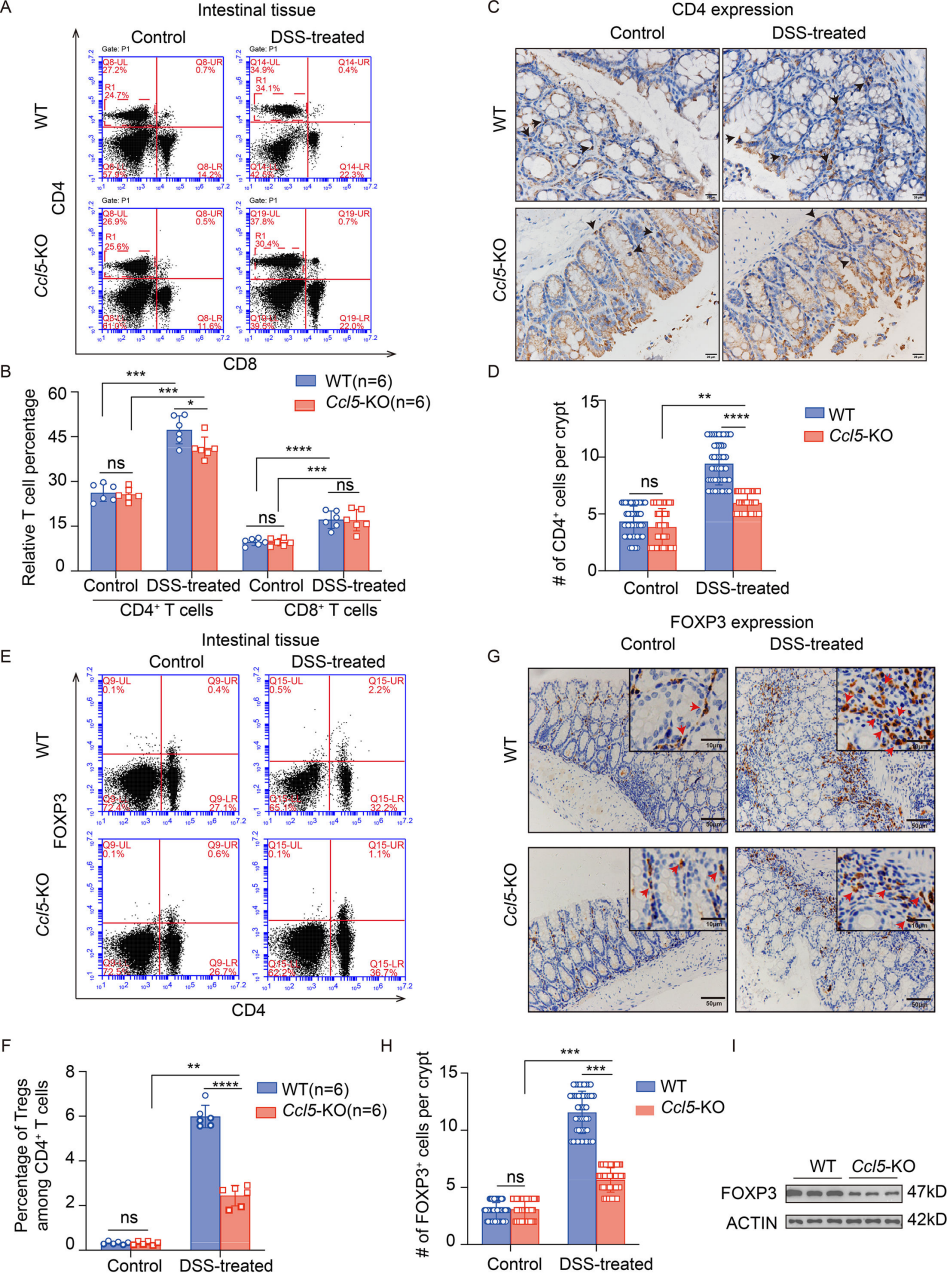
C-C motif chemokine ligand 5 (CCL5) deficiency impairs CD4^+^ T cell activity by reducing the number of forkhead box protein 3 (FOXP3^+^) regulatory T cells (Tregs) in the inflamed intestine. (**A and B**) Flow cytometric analysis of number of CD4^+^ and CD8^+^ T cells in intestinal lymphoid tissues from mice with indicated genotypes 4 days after 7-day 2.5% dextran sulfate sodium salt (DSS) administration. Graphs (**B**) show the relative percentage of, respectively, CD4^+^ and CD8^+^ T cells (*n* = 6 per group). (**C and D**) Representative CD4 staining of distal colon sections from wildtype (WT) and CCL5 knockout (*Ccl5*-KO) mice 4 days after 7-day 2.5% DSS administration (**C**; scale bars, 20 μm), with quantitative analysis (**D**, *n* = 50); black arrows indicate CD4-positive cells. (**E and F**) Flow cytometric plots (**E**) of FOXP3^+^ CD4^+^ T cell population in intestines from control or DSS-treated mice with indicated genotypes (*n* = 6 per group). Percentage of FOXP3^+^ population among CD4^+^ T cells is shown (**F**, *n* = 6). (**G and H**) Representative FOXP3 staining of distal colon sections from WT and *Ccl5*-KO mice 4 days after 7-day 2.5% DSS administration (**G**; scale bars: 10 μm, 50 μm), with quantitative analysis (**H**, *n* = 50); red arrows indicate FOXP3-positive cells. (**I**) Immunoblotting of FOXP3 expression in colonic tissues from WT and *Ccl5*-KO mice 4 days after 7-day 2.5% DSS administration. Results shown are the mean ± SEM (ns, nonsignificant; *P<0.05, **P<0.01, ***P<0.001, ****P<0.0001) of triplicate determination from three independent experiments.

Previous studies have demonstrated that CCL5 exerts its effects through its receptors CCR5 and CCR1 on CD4^+^ T cells, regulating immune responses and inflammatory reactions, and playing a crucial role in the immune environment of HIV infection, autoimmune diseases, and tumors [28]. We first isolated the intestine-infiltrated immune cells and analyzed the amount of total CD4^+^ and CD8^+^ T cells by flow cytometry. Results indicated that although intestinal inflammation following DSS treatment resulted in increased levels of both CD4^+^ and CD8^+^ T cells, the increase in the percentage of CD4^+^ T cells was significantly lower in the *Ccl5*-KO group than in the WT group (Figure 3A and B, Supplementary Figure 1A–C). Subsequent histological findings were consistent with the flow cytometric data: an appreciable increase in CD4 expression was observed in both WT and *Ccl5*-KO mice; however, the magnitude of increase was significantly less in *Ccl5*-KO mice compared with WT mice (Figure 3C and D).

The activation and regulation of different T cell subsets are governed by a complex network of transcription factors and intercellular signaling. Specifically, the transcription factor forkhead box P3 (Foxp3) is a critical marker and functional maintainer of Tregs, with its expression closely associated with the development and function of Tregs [29]. Further flow cytometric analysis revealed a significant reduction in the proportion of FOXP3-positive cells within the CD4^+^ T cell subsets (Figure 3E and F, Supplementary Figure 1A–C). Additionally, the IHC and WB analysis of FOXP3 in the intestines of both groups under DSS treatment corroborated these findings (Figure 3G–I). Subsequently, we initially analyzed whether the number of non-T cells in peripheral blood and spleen exhibited any significant changes. Flow cytometric analysis of CD11b, a marker commonly used to identify monocytes, macrophages, neutrophils, and NK cells, revealed no significant differences in the number of CD11b^+^ cells between WT and *Ccl5*-KO mice, both under normal physiological conditions and during DSS-induced acute colitis (**Supplementary Figure 2A and B**). Interestingly, the number of CD4^+^ T cells in both peripheral blood and spleen did not show significant differences, in contrast with the changes observed in the intestines. Following this, we further assessed the number of Tregs in the spleen and peripheral blood of both WT and KO mice with UC. Unexpectedly, flow cytometric results revealed no significant differences between the two groups (**Supplementary Figure 2C and D**), suggesting that the migration ability of Tregs to inflamed sites remained unaffected. Le et al. [30] demonstrated that CCL5 not only enhances the proliferative capacity of Tregs but also boosts their ability to secrete immunosuppressive cytokines such as IL-10. Therefore, these results suggest that CCL5 plays a crucial role in regulating the number of Tregs within the intestines, thereby modulating the function of CD4^+^ T cells in an inflammatory environment and consequently affecting the severity of intestinal inflammation.

### CCL5 deficiency in nonimmune cells suppresses IL-33 level via inhibiting NF-κB signaling

**Figure 4:**
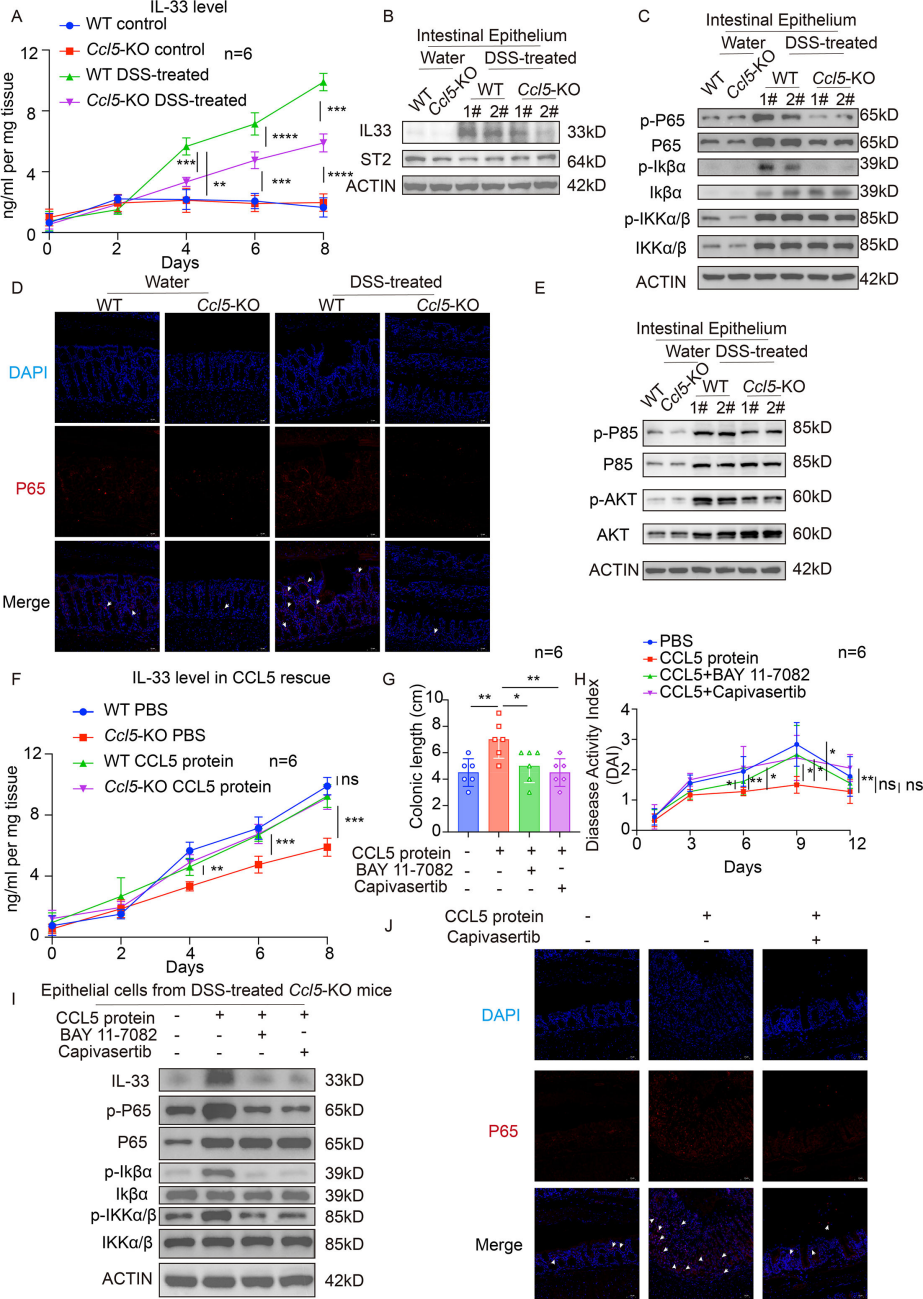
C-C motif chemokine ligand 5 (CCL5) deficiency in nonimmune cells suppresses interleukin-33 (IL-33) level via inhibiting nuclear factor kappa B (NF-κB) signaling. (**A**) Enzyme-linked immunosorbent assay (ELISA) analysis of the level of IL-33 in colon explant cultures at the indicated time points (days 0, 2, 4, 6, 8) during the 7-day dextran sulfate sodium salt (DSS) treatment in wildtype (WT) and CCL5 knockout (*Ccl5*-KO) mice (*n* = 6 per group). (**B**) Immunoblotting of IL-33 and ST2 in intestinal epithelial cells of WT and *Ccl5*-KO mice 4 days after 7-day 2.5% DSS administration. (**C**) Immunoblotting of NF-κB (**P65**) related pathway in intestinal epithelium of WT and *Ccl5*-KO mice 4 days after 7-day 2.5% DSS administration. (**D**) Immunofluorescence staining for P65 (red) in colonic sections of WT and *Ccl5*-KO mice 4 days after 7-day 2.5% DSS administration; DNA (DAPI, blue); scale bars, 50 μm. White arrows indicate P65-positive cells with nuclear translocation. (**E**) Immunoblotting of PI3K/Akt pathway in intestinal epithelium of WT and *Ccl5*-KO mice 4 days after 7-day 2.5% DSS administration. (**F**) ELISA analysis of IL-33 levels in colon explant cultures of WT and *Ccl5*-KO mice at the indicated time points (days 0, 2, 4, 6, 8) during 7-day DSS treatment with CCL5 small protein interventions (*n* = 6 per group). (**G**) Colon length measurements on day 12 in *Ccl5*-KO mice treated with different drug groups (control, CCL5 small protein, CCL5 small protein + BAY 11-7082, CCL5 small protein + Capivasertib); *n* = 6 per group. (**H**) Recording of DAI different time points (**D0, D3, D6, D9, D12**) in different drug treatment groups during the treatment period. (**I**) Immunoblot analysis of corresponding protein levels in the intestinal epithelial tissues of *Ccl5*-KO mice after treatment with different drug groups. (**J**) Immunofluorescence staining analysis of P65-positive (red) cells in the intestines of *Ccl5*-KO mice treated with different drug groups (control, CCL5 small protein, CCL5 small protein + Capivasertib); DNA (DAPI, blue), scale bars, 50 μm. White arrows indicate P65-positive cells with nuclear translocation. Results shown are the mean ± SEM (ns, nonsignificant; *P<0.05, **P<0.01, ***P<0.001, ****P<0.0001) of triplicate determination from three independent experiments.

Recent studies in humans have observed the essential role of high levels of IL-33 in modulating Th1/Th2-derived inflamed lesions of IBD patients, implying IL-33 plays a critical role in regulating T cell activity in disease pathogenesis [31,32]. Initially, we assessed the differences in IL-33 levels in the intestinal tissues of WT and *Ccl5*-KO mice during DSS induction. As anticipated, IL-33 levels significantly increased on days 2–3 of DSS treatment in both groups. However, by around day 4, the increase in IL-33 levels showed a marked difference between the *Ccl5*-KO and WT groups (Figure 4A). Concurrently, WB analysis (Figure 4B) revealed that IL-33 protein levels in the intestinal epithelial cells of *Ccl5*-KO mice were lower than those in the WT group following DSS treatment. Previous studies demonstrated that IL-33 could bind its receptor ST2 on Tregs to enhance TGF-β1-mediated differentiation of Tregs and recruit these cells into inflamed tissues [33]. There were no significant differences in the expression levels of the primary receptor ST2 in the intestinal epithelial cells across the groups (Figure 4B).

Numerous studies have highlighted the critical role of the NF-κB signaling pathway in regulating IL-33 levels. In the study conducted by Jin et al. [34], it was demonstrated that LPS activates the NF-κB (P65) subunit, which subsequently drives the up-regulation of IL-33 in alveolar epithelial cells. Additionally, the use of NF-κB (P65) inhibitors, such as BAY 11-7082, was shown to restore IL-33 expression in LPS-stimulated epithelial cells. Furthermore, in dendritic cells, flagellin triggers the MyD88/NF-κB signaling cascade, resulting in increased IL-33 production. Inhibition of TLR5 or NF-κB activation through quinazoline significantly attenuated these effects, including the reduction in IL-33 production. To investigate the role of NF-κB-related signaling pathways in the regulation of IL-33 levels in our model, we further analyzed changes in the NF-κB (P65) protein levels in epithelial cells after DSS treatment. The results indicated that under DSS treatment, *Ccl5* deficiency not only inhibited the protein levels of NF-κB (P65) itself, but also led to a corresponding decrease in the phosphorylation levels of NF-κB (P65), which reflect its activation (Figure 4C).

Numerous NF-κB activation pathways have been identified, all of which depend on sequentially activated kinase cascades. These extracellular signals activate the IKK complex, which phosphorylates IκBα, leading to its ubiquitin-mediated degradation. The released NF-κB then translocates into the nucleus, where it promotes NF-κB-dependent transcription [35]. WB analysis further indicated that *Ccl5* deficiency suppressed IκBα phosphorylation (Figure 4C). Subsequently, immunofluorescence staining results confirmed that the absence of CCL5 led to the attenuation of NF-κB translocation (Figure 4D). To further investigate the impact of nonimmune cell-derived CCL5 on IL-33 levels through regulation of NF-κB activity, we conducted rescue experiments using CCL5 protein and P65 inhibitors, BAY 11-7082 in the DSS mouse model (**Supplementary Figure 3A**). The results confirmed that CCL5 protein treatment successfully reversed the decreased IL-33 levels in the intestinal tissues of *Ccl5*-KO mice after DSS treatment (Figure 4F). As expected, CCL5 protein treatment significantly reversed the colitis phenotype in mice, while the addition of BAY 11-7082 markedly diminished the therapeutic effects of CCL5 protein treatment (Figure 4G and H and **Supplementary Figure 3B and D)**. Meanwhile, WB analysis of the intestinal epithelial cells from DSS-treated *Ccl5*-KO mice after different drug treatments further confirmed that *in vivo* CCL5 protein therapy could reverse the changes in IL-33 levels in nonimmune cells, while BAY 11-7082 counteracted this effect (Figure 4I). Additionally, CCL5 protein therapy was able to reverse the trends and phosphorylation levels of IκBα/NF-κB caused by *Ccl5* deficiency, a finding corroborated by the immunofluorescence staining results (Figure 4J).

### PI3K/Akt pathways are involved in CCL5-induced NF-κB activity

Existing studies have shown that the PI3K/Akt pathway is a key signaling cascade that mediates the activation of the NF-κB pathway in human cancer cells [36]. To investigate the effect of CCL5 on PI3K/Akt pathway activity, we further analyzed the changes in pathway protein levels in the intestinal epithelium of WT and *Ccl5*-KO mice between the DSS treatment and control groups. The results indicated that *Ccl5* deficiency led to a significant decrease in the phosphorylation levels of the PI3K/Akt pathway, suggesting that the loss of CCL5 inhibits PI3K/Akt pathway activity, which is consistent with the previously observed suppression of NF-κB activity (Figure 4E). To validate this correlation, we further treated DSS model mice with an Akt inhibitor, Capivasertib (AZD5363) (**Supplementary Figure 3A**). Consistent with the effect of the BAY 11-7082, Akt inhibition reversed the therapeutic effect of CCL5 protein treatment on the colitis phenotype (Figure 4G and H and **Supplementary Figure 3B)**. WB analysis also confirmed that Akt inhibition counteracted the CCL5 protein treatment’s effect on IL-33 levels, while the phosphorylation levels of IκBα/NF-κB were suppressed (Figure 4I). Additionally, immunofluorescence staining results indicated that the restoration of P65 translocation following CCL5 treatment was once again inhibited (Figure 4J). Taken together, these findings suggest that the deficiency of CCL5 in nonimmune cells impairs IL-33-induced Treg differentiation by inhibiting the PI3K/Akt/NF-κB (P65) signaling pathway.

### CCL5 deficiency impedes IL-33-induced Treg formation via JAK1/STAT5 signaling pathway

**Figure 5:**
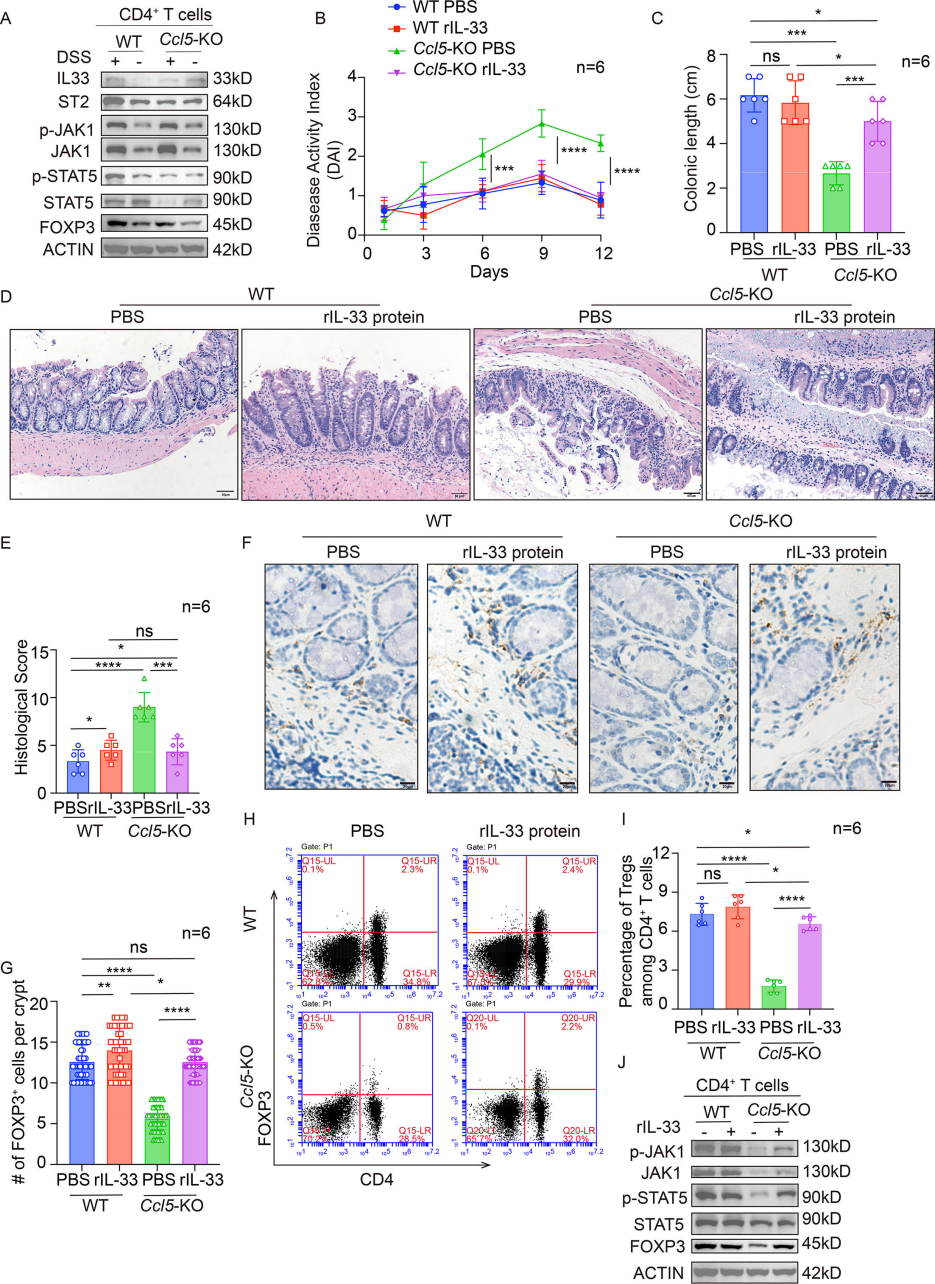
C-C motif chemokine ligand 5 (CCL5) deficiency impedes interleukin-33 (IL-33)-induced regulatory T cell (Treg) formation via JAK1/STAT5 signaling pathway. (**A**) Immunoblotting of IL-33, ST2, forkhead box protein 3 (FOXP3), and JAK1/STAT5 signaling in intestinal CD4^+^ T cells of wildtype (WT) and CCL5 knockout (*Ccl5*-KO) mice 4 days after 7-day 2.5% dextran sulfate sodium salt (DSS) administration. (**B and C**) Mice received daily intraperitoneal injections of rIL-33 protein (10 ng/µL) at the onset of the DSS regimen. The intraperitoneal administration of different therapeutic agents continued for an additional 4 days after 7-day DSS treatment. Subsequently, the mice were killed, and the affected intestinal tissues were analyzed. Disease activity index (DAI) (**B**) and colon length (**C**) were monitored, *n* = 6 per group. (**D and E**) Representative H&E staining (**D**) of colon sections in mice from different treated groups (scale bars, 50 μm). Histological score (**E**) was quantified. (**F and G**) Representative FOXP3 staining of distal colon sections from rescued DSS-treated mice with indicated genotypes after 8-day rIL-33/PBS treatment (**F**; scale bars, 20 μm) and quantitative analysis (**G**, *n* = 50). (**H and I**) Flow cytometric plots (**H**) of FOXP3^+^ CD4^+^ T cell population in intestines from rescued DSS-treated mice with indicated genotypes after 8-day rIL-33/PBS treatment and quantitative analysis (**I**, *n* = 6). (**J**) Immunoblotting of FOXP3 and JAK1/STAT5 signaling in intestinal CD4^+^ T cells, respectively, from rescued DSS-treated mice with indicated genotypes after 8-day rIL-33/PBS treatment. The results are shown as the mean ± SEM (ns, nonsignificant; *P<0.05, **P<0.01, ***P<0.001, ****P<0.0001) of triplicate determination from three independent experiments, with statistical significance assessed using one-way analysis of variance (ANOVA) followed by Tukey’s post hoc test.

Subsequently, to investigate the specific mechanism by which CCL5 co-operates with changes in IL-33 levels to regulate Treg levels, we further isolated CD4^+^ T cells from intestinal tissues and performed WB analysis (Figure 5A). The results revealed no significant differences in IL-33 or its receptor ST2 protein levels between the WT and *Ccl5*-KO groups under DSS induction. However, FOXP3 protein levels were notably reduced in the *Ccl5*-KO group. As expected, WB analysis also confirmed the inactivation of the downstream JAK1/STAT5 signaling pathway in CD4^+^ T cells from the *Ccl5*-KO group (Figure 5A).

To further investigate the role of IL-33 in regulating the downstream JAK1/STAT5 signaling pathway and its impact on Treg formation, we intraperitoneally injected 100 µL of recombinant IL-33 protein (rIL-33, HY-P7218, MCE, 10 ng/µL) into both WT and *Ccl5*-KO mice daily for 7 days following 2.5% DSS induction, as previous results indicated that IL-33 levels primarily differed on day 4 (**Supplementary Figure 4A**). Notably, timely rIL-33 treatment effectively reversed the phenotypic differences observed between *Ccl5*-KO and WT mice under DSS induction. According to DAI records (Figure 5B), the severity of colitis and intestinal shortening in *Ccl5*-KO mice (Figure 5C and **Supplementary Figure 4B**) was significantly alleviated under rIL-33 treatment, findings that were corroborated by H&E staining results across different groups (Figure 5D and E). To examine the effect of rIL-33 on FOXP3 expression in the intestines of *Ccl5*-KO mice, we performed FOXP3 IHC staining on tissue samples from different groups (Figure 5F and G). rIL-33 treatment was found to effectively reverse the reduced FOXP3 expression caused by CCL5 deficiency. This observation was further supported by flow cytometric analysis of FOXP3^+^ cells in the intestine (Figure 5H and I and **Supplementary Figure 4C)**. Subsequently, we isolated CD4^+^ T cells from the intestines of different groups after rIL-33 treatment and performed WB analysis (Figure 5J), which revealed that rIL-33 significantly increased JAK1/STAT5 signaling pathway activity and, correspondingly, enhanced FOXP3 expression in the *Ccl5*-KO group. Additionally, consistent with our previous findings, the suppressed state of the downstream NF-κB (P65) signaling pathway in the *Ccl5*-KO group was not reversed by rIL-33 treatment (**Supplementary Figure 4D**). In summary, these results demonstrate that CCL5 deficiency impairs Treg formation by suppressing IL-33 levels through the JAK1/STAT5 pathway in CD4^+^ T cells.

### Expression of CCL5 is positively correlated with expression of FOXP3 in clinic

**Figure 6:**
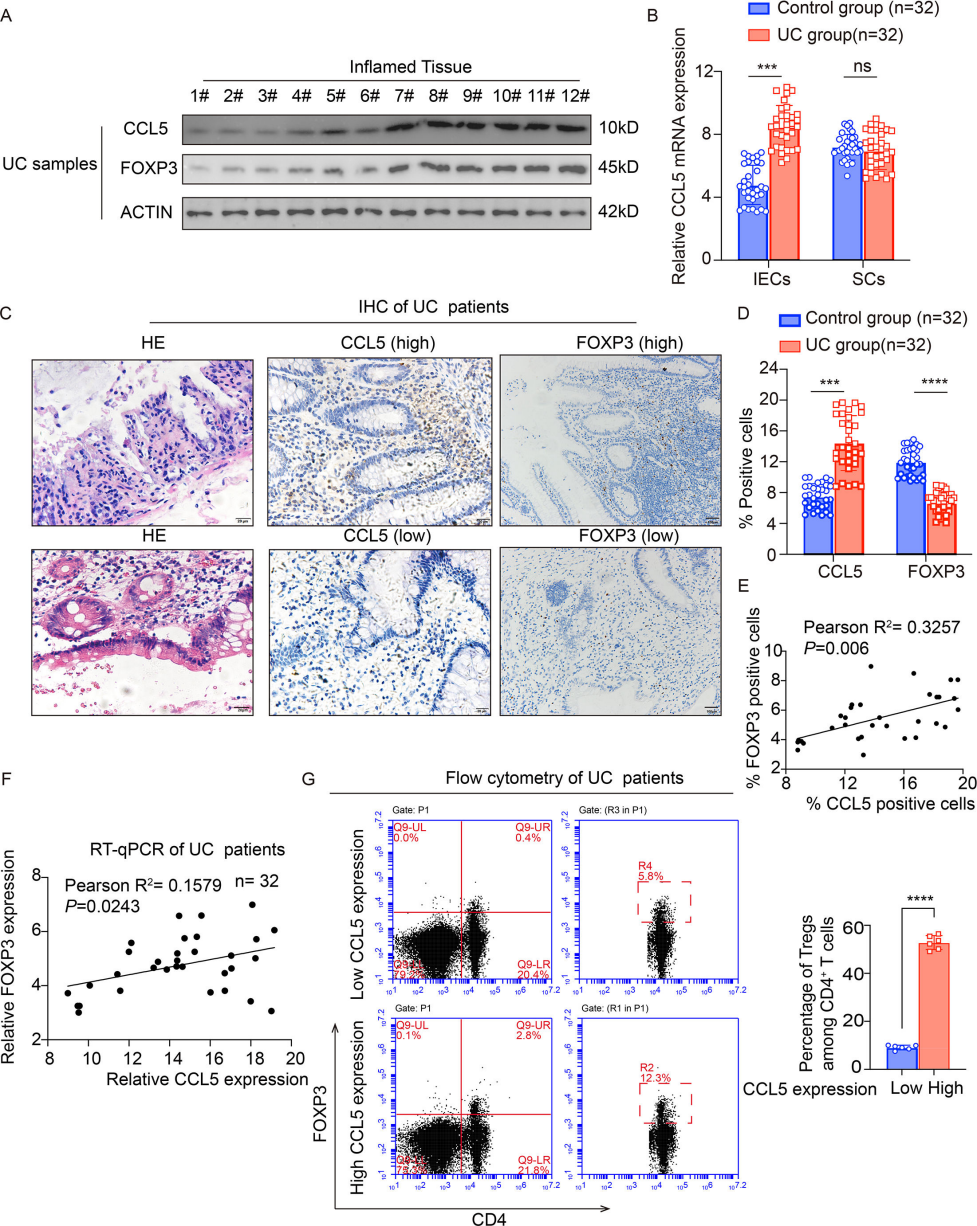
Expression of C-C motif chemokine ligand 5 (CCL5) is positively correlated with expression of forkhead box protein 3 (FOXP3) in clinic. (**A**) Immunoblotting (*n* = 12) of CCL5 and FOXP3 expression in inflamed tissue in ulcerative colitis (UC) patients. (**B**) Quantitative polymerase chain reaction (qPCR) analysis (*n* = 32) of CCL5 expression, respectively, in intestinal epithelial (IECs) and stromal cells (SCs) from inflamed and adjacent normal tissue in UC patients. (**C and D**) Representative H&E staining (right), CCL5 (middle), and FOXP3 (left) staining of inflamed intestinal tissue from UC patients with different levels of CCL5 expression (CCL5 [high] vs. CCL5 [low]), (**C**; scale bars, 20 μm, 100 μm) and quantitative analysis of CCL5 and FOXP3 expression (**D**, *n* = 32 per group) in inflamed (UC) and adjacent normal tissue (NC). (**E**) Correlation analysis between the proportion of CCL5-positive cells and FOXP3-positive cells in inflamed (UC) tissue from UC patients (*n* = 32). (**F**) Quantitative real-time polymerase chain reaction (RT-qPCR) analysis of the correlation between CCL5 and FOXP3 expression levels in inflamed intestinal tissue (*n* = 32) from UC patients with different levels of CCL5 expression. (**G**) Flow cytometric plots (right) of FOXP3^+^ CD4^+^ T cell population in intestines from CCL5-low and CCL5-high UC patient tissues with quantitative analysis (left, *n* = 6). Results shown are the mean ± SEM (ns, nonsignificant; ***P<0.001, ****P<0.0001) of triplicate determination from three independent experiments.

To validate the correlation between CCL5 expression levels and the number of Tregs in a clinical setting, we obtained inflamed intestinal tissues from UC patients, and subsequent WB analysis revealed a positive correlation between the expression levels of CCL5 and FOXP3 in these tissues. Notably, UC patients exhibiting reduced CCL5 expression in their inflamed intestinal tissues also demonstrated a corresponding decrease in FOXP3 expression (Figure 6A). Furthermore, after separating intestinal epithelial cells from stromal cells and analyzing them, we found that the differential expression of CCL5 was primarily localized within the epithelial cells (Figure 6B). This finding suggested a specific role for CCL5 in epithelial cells in responding to intestinal inflammation. Whereafter, we obtained tissues from paraffin-embedded clinical samples of UC patients to assess CCL5 and FOXP3 expression levels using IHC staining. The results indicated that in patients with high CCL5 expression, the level of FOXP3 expression in the intestinal tissue was also significantly elevated (Figure 6C–E). Additionally, quantitative PCR (qPCR) analysis using patient tissues, isolated intestinal villus tissues (intestinal epithelial cells), and intestinal stromal tissues (intestinal stromal cells), respectively, revealed that in intestinal epithelial cells, transcription levels of CCL5 and FOXP3 coincided with a trend of positive correlation (*n* = 32, *R* ^2^ = 0.1579, *P*=0.0243) (Figure 6F). To further validate the expression relationship between the two, the results of flow cytometric analysis verified that in patients, the expression levels of CCL5 and FOXP3 were positively correlated (Figure 6G and **Supplementary Figure 5A and B**). In sum, results indicate that CCL5 plays a crucial role in the intestinal response to inflammation in clinical contexts, and its expression level positively correlates with that of FOXP3 in the intestine.

## Discussion

This study identifies CCL5 as a critical regulator of FOXP3^+^ Treg formation by modulating IL-33 levels, thereby synergistically influencing the regulation of the JAK1/STAT5 signaling pathway in CD4^+^ T cells, which governs the formation of FOXP3^+^ T cells. Consequently, CCL5 plays a pivotal role during acute colitis. This also suggests that CCL5 plays different roles at various stages of colitis. Analysis of clinical data reveals that in UC patients, CCL5 expression in inflamed intestinal tissues is significantly higher than in corresponding normal tissues. Moreover, the expression levels of CCL5 and FOXP3 are positively correlated within the intestinal environment. Using a combination of *in vivo* drug treatments and a DSS-induced acute colitis model, we further established the protective role of CCL5 in acute colitis. Not only does nonimmune cell-derived CCL5 influence intestinal IL-33 levels through the PI3K/Akt/NF-κB (P65) pathway, but CCL5 also co-operates with IL-33 to affect downstream Treg activity. These findings provide further insight into the varying roles of CCL5 at different stages of colitis, highlighting its potential as a therapeutic target, which warrants further exploration.

CCL5, a critical chemokine, plays a multifaceted and dual role in immune and inflammatory responses, and its involvement in IBD has been increasingly recognized [28]. Previous studies have shown that CCR5, the receptor for CCL5, plays a role in mediating inflammation in IBD. CCR5-deficient (CCR5^-/-^) mice exhibit reduced leukocyte recruitment to the colon and show attenuation of inflammation in colitis models. This suggests that CCR5 and its ligand CCL5 are involved in promoting inflammation and that targeting CCR5 could offer therapeutic benefits in IBD [12]. However, in our study, we observed that CCL5 deficiency exacerbates colitis during the acute phase, which contrasts with the findings in chronic inflammation. This discrepancy highlights the complex, context-dependent role of CCL5 in IBD, with potentially protective effects during the acute phase of inflammation and pro-inflammatory effects during chronic stages. Our findings indicate that CCL5, derived from both immune and nonimmune cells in the intestine, plays a complex role that depends on the context of the inflammatory phase. Specifically, during acute colitis, CCL5 appears to exert protective effects by modulating immune cell recruitment and tissue damage [37]. In contrast, in chronic phases of inflammation, CCL5’s role may be less protective. These observations highlight the importance of considering the temporal dynamics of CCL5 expression in relation to disease progression and suggest that its effects on intestinal inflammation are context-dependent, warranting further investigation. Additionally, consistent with prior findings, CCL5 levels in UC patients’ inflamed intestinal tissues were significantly elevated, which further supports the hypothesis that CCL5 plays a key role in regulating the inflammatory response in IBD.

It is noteworthy that while previous studies generally consider CCL5 to have a pro-inflammatory role in IBD, particularly in exacerbating disease severity during the chronic inflammation phase, our findings diverge in the acute colitis model. We observed that CCL5 deficiency in the acute phase actually exacerbated the intestinal inflammation phenotype, suggesting a potential protective role for CCL5 during the acute phase. Several factors may contribute to this result. First, existing studies have indicated that CCL5 from nonimmune cells may play a role in immune regulation. In addition to its production by traditional immune cells, nonimmune cells, such as epithelial cells, endothelial cells, and fibroblasts, can also produce CCL5 in local inflammatory responses, working in concert with immune cells to regulate the balance of immune responses [38,39]. In our model, this nonimmune cell-derived CCL5 may have played a regulatory role during the acute phase. The synergistic action of CCL5 and IL-33 may influence the composition and activity of immune cells, playing a positive role early in inflammation, thereby limiting excessive immune responses and preventing the expansion of inflammation.

Second, the dual role of CCL5 in acute and chronic inflammation may also explain the discrepancy between our results and those of other studies. Existing studies have revealed that CCL5 exhibits a dual role in different immune environments and pathological stages [40–43]. During acute infection or inflammation, CCL5 aids in the early recruitment of immune cells and local responses, thereby effectively controlling the condition. However, in chronic inflammation, excessive expression of CCL5 may promote the continuous infiltration of immune cells, leading to chronic inflammation and immune pathological responses [44]. Therefore, in the acute colitis model, CCL5 may help limit the expansion of lesions through its pro-inflammatory effects, while in the chronic phase, it may transition to a factor that exacerbates immune damage. Finally, the differences in CCL5’s downstream receptors may explain its varied functions in different immune cells and tissues. By binding to receptors such as CCR1, CCR3, and CCR5, CCL5 regulates the recruitment and activation of immune cells [45]. Due to the differential distribution and functions of these receptors, the role of CCL5 on various cells may determine its dual nature in immune responses. This could be one of the reasons why we observed an exacerbation of the acute colitis phenotype in the absence of CCL5 in our experiments. Therefore, the role of CCL5 in immune regulation is not only dependent on the timing and context of its expression but also influenced by the distribution of its receptors and the types of cells involved. Future research should further explore its specific functions and mechanisms in different types of immune responses and investigate the precise mechanisms of CCL5 at different stages, enabling the development of corresponding therapeutic strategies tailored to the disease stage in clinical practice.

CCL5 regulates immune responses through interactions with various immune factors. As an important chemokine, CCL5 not only participates in T cell recruitment via the CCR5 receptor, but also modulates T cell proliferation, differentiation, and activation through synergistic interactions with other immune factors [4]. IL-12, a critical Th1 cytokine, enhances CCL5’s effects on T cells by promoting CCR5 expression. Studies have demonstrated that the interaction between CCL5 and IL-12 fosters T cell differentiation into Th1 cells, thereby amplifying Th1 immune responses, especially during immune reactions against viral and bacterial infections. Through the CCL5-CCR5 pathway, CCL5 not only promotes T cell activation but also influences the polarization of immune responses via IL-12, playing a crucial role in the immune defense [46,47]. In addition to IL-12, the interaction between CCL5 and CXCL12 (SDF-1) is pivotal in the functional regulation of T cells. CXCL12 activates T cells via its receptor CXCR4, regulating immune cell migration and localization. The synergistic action of CCL5 and CXCL12 enhances T cell activation and migration, thereby strengthening immune responses [48,49]. It has been reported that the IL-1 family member IL-33, constitutively expressed in epithelial cells at barrier sites [50,51], stimulates Treg responses by providing necessary signals for Treg accumulation and maintenance in inflamed tissues [33]. Recent studies have shown high levels of IL-33 in inflamed lesions of IBD patients, suggesting its involvement in disease pathogenesis [31,32,52,53]. Our research focuses on how CCL5 influences the formation of FOXP3^+^ T cells induced by IL-33 in intestinal epithelial cells during inflammatory responses, thereby affecting the function of CD4^+^ T cells. *In vivo* rescue experiments using CCL5 protein and P65 inhibitors confirmed that IL-33 levels in *Ccl5*-KO mice were significantly restored by CCL5 protein rescue. However, this restoration was negated when P65 inhibitors and AKT inhibitors were used. Our findings indicated that, in an acute inflammation model, nonimmune cell-derived CCL5 interacted with IL-33 to regulate the composition and activity of immune cells, affecting T cell immune function. Moreover, the regulation of IL-33 levels by CCL5 was mediated through the PI3K/Akt/P65 signaling pathway.

CCL5 also plays a crucial regulatory role in the Jak-Stat signaling pathway, significantly affecting T cell function. Through its receptor CCR5, CCL5 activates Jak2 and Jak3, which subsequently phosphorylate and dimerize transcription factors such as Stat1, Stat3, and Stat5, ultimately affecting gene expression. Through this process, CCL5 not only promotes T cell proliferation but also regulates various aspects of T cell differentiation, immune response polarization, and apoptosis [54]. Our research further highlights the role of nonimmune cell-derived CCL5 in regulating the Jak-Stat signaling pathway in T cells. We observed that in the DSS-induced acute colitis model, the activation of the JAK1/STAT5 signaling pathway in CD4^+^ T cells from *Ccl5*-KO mice was significantly suppressed, and corresponding FOXP3 levels were also reduced. To validate the role of CCL5 in conjunction with IL-33 in regulating FOXP3 expression through the JAK1/STAT5 signaling pathway, we performed rescue experiments using rIL-33 protein in *Ccl5*-KO mice. The results showed that rIL-33 not only alleviated the exacerbated colitis phenotype in *Ccl5*-KO mice but also reversed the reduction in FOXP3^+^ CD4^+^ T cell numbers in the intestinal tissue through rescuing JAK1/STAT5 signaling pathway. This provides new insights into the multifaceted role of nonimmune cell-derived CCL5 in immune responses. Through this mechanism, CCL5 not only regulates T cell activation but also, through its interaction with IL-33, governs T cell differentiation and immune tolerance, highlighting its potential role in immune balance.

In summary, our study highlights the essential role of CCL5 in regulating Treg formation and its involvement in the inflammatory response during colitis. We show that CCL5 modulates IL-33 levels and activates the JAK1/STAT5 pathway in CD4^+^ T cells, playing a protective role in acute colitis. Interestingly, while CCL5 has a protective effect in the early stages of inflammation, it may contribute to disease exacerbation in chronic inflammation. Our findings suggest that CCL5 acts contextually, with different functions at various stages of the disease. The significant elevation of CCL5 levels in the inflamed tissues of UC patients further underscores its potential as a therapeutic target in IBD, particularly for managing acute phases of the disease.

**Clinical Perspectives**

- CCL5, as a critical chemokine, plays a complex and dual role in immune and inflammatory responses in UC, although the exact underlying mechanism remains unclear.
- CCL5 knockout exacerbates acute DSS-induced colitis by impairing IL-33 levels through disruption of the PI3K/Akt/NF-κB signaling pathway and negatively affecting FOXP3^+^ Treg formation via disruption of the JAK1/STAT5 pathways, thereby enhancing intestinal inflammation.
- CCL5 correlates with FOXP3 in UC patients, emphasizing its potential as both a biomarker and a therapeutic target for UC treatment.

## Supplementary material

online supplementary material 1.

## Data Availability

The datasets used and/or analyzed during the current study are available from the corresponding author on reasonable request.

## Open Access

This article has been published open access under our Subscribe to Open programme, made possible through the support of our subscribing institutions, learn more here: https://portlandpress.com/pages/open_access_options_and_prices#conditional

## Abbreviations

CCL5: C-C chemokine motif ligand 5
DSS: dextran sulfate sodium salt
FoxP3: forkhead box protein P3
IBD: inflammatory bowel disease
IL-33: interleukin 33
Tregs: regulatory T cells
UC: ulcerative colitis
