# Supplementary material for: CCL5 deficiency aggravates acute DSS-induced colitis by restricting IL-33-induced formation of Tregs in intestinal tract
Source: Clin Sci (Lond). 2026 Jan 7;140(1):27–46. doi: 10.1042/CS20256734 (PMC12862953; doi:10.1042/CS20256734)
Supplement: online supplementary material 1. [file cs-140-1-CS20256734-s001.docx]

**
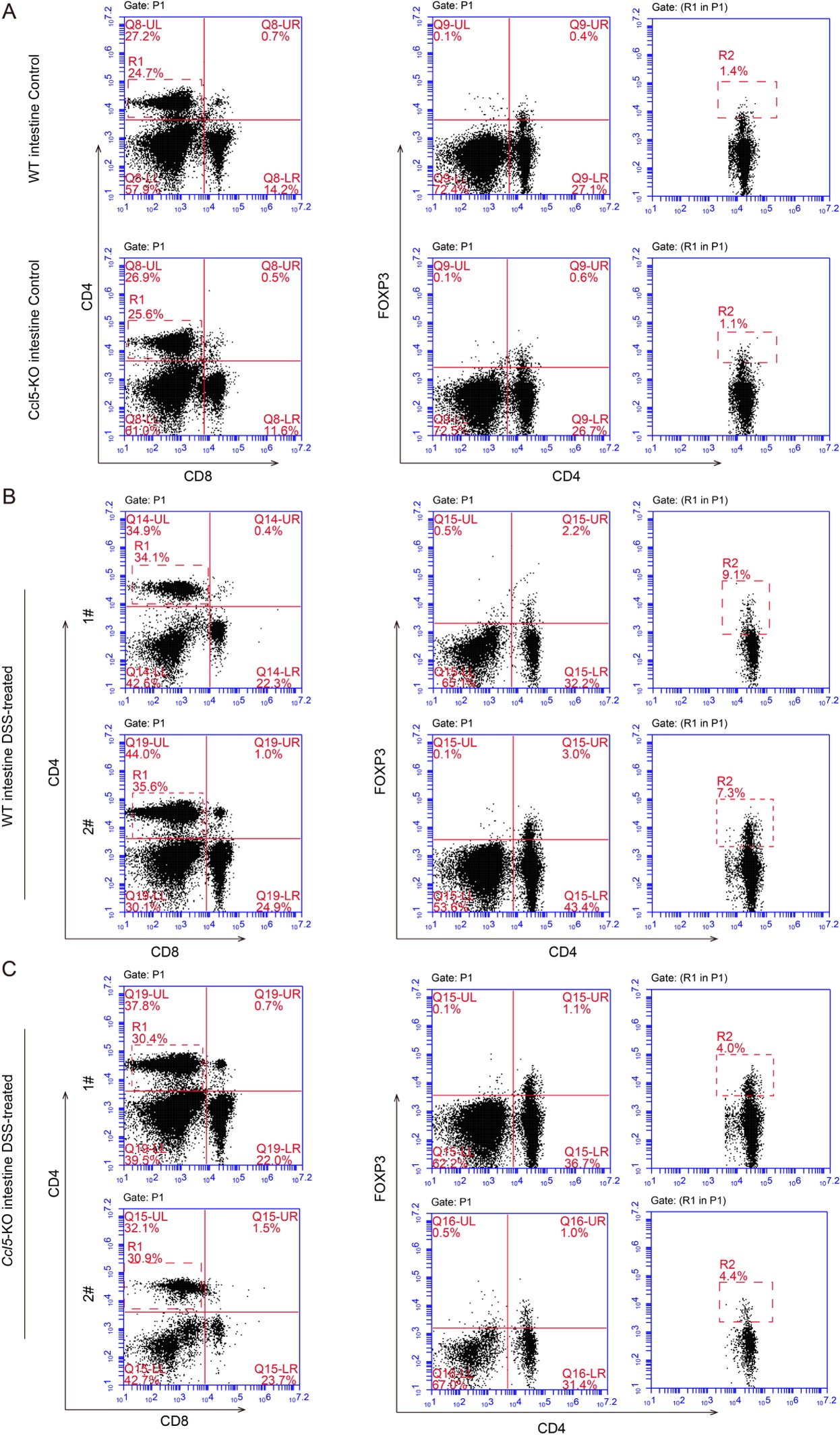
**

**Supplementary Figure 1:** (A-C) Flow cytometry analysis of FOXP3^+^ CD4^+^ T cell numbers in the intestinal lymphoid tissues of WT and *Ccl5*-KO mice after 7-day 2.5% DSS treatment.


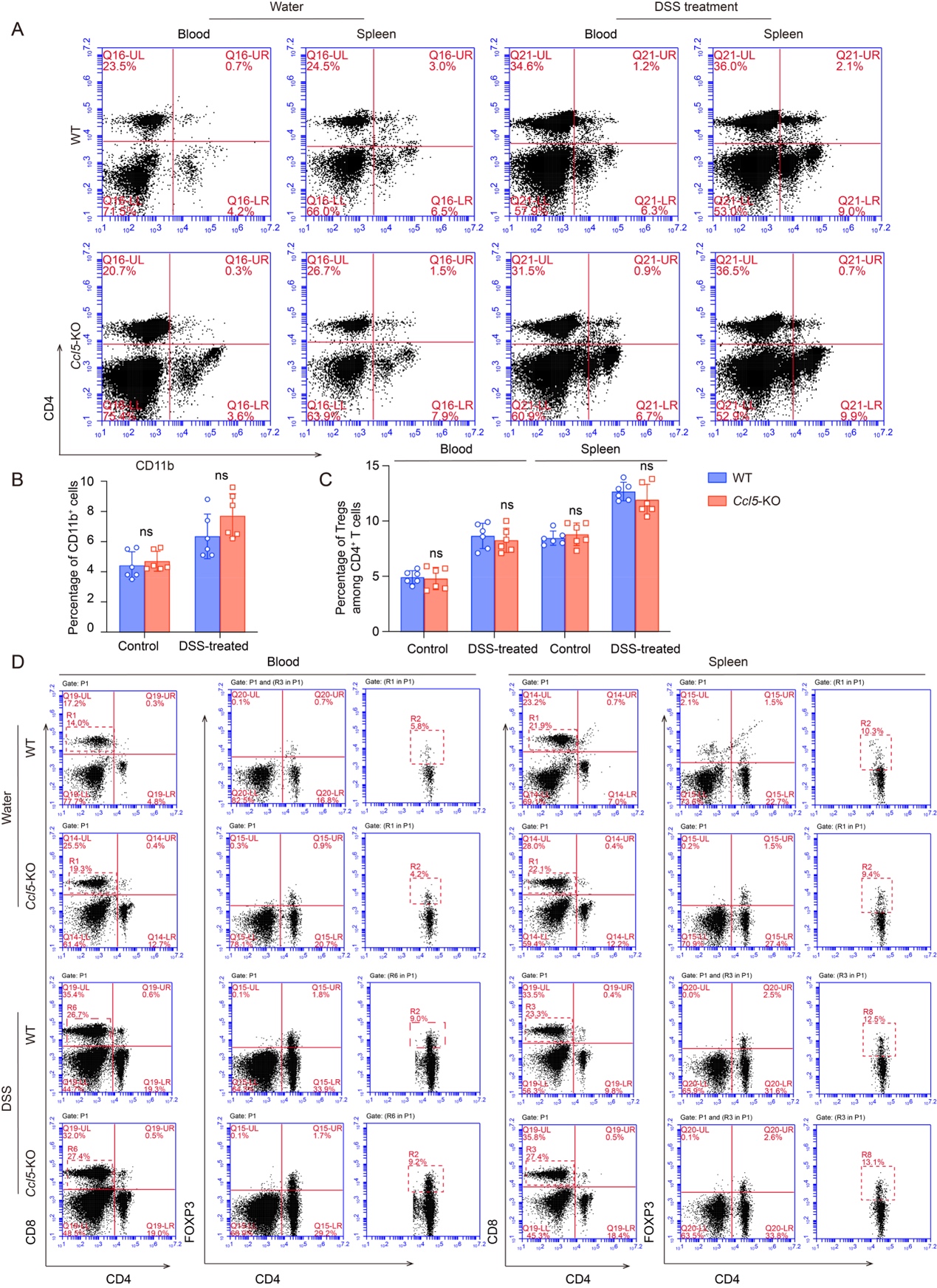
**Supplementary Figure 2:**

(A and B) Flow-cytometric plots of CD4^+^ and CD11b^+^ cells respectively in spleen and peripheral blood of WT and *Ccl5*-KO mice after 7-day 2.5% DSS treatment(A) and quantitative analysis of CD11b^+^ populations [(B), n=6 per group].

(C and D) The percentage of Treg cells in spleen and peripheral blood were analyzed by flow cytometry (D). The total number of FOXP3^+^ T cells in spleen and peripheral blood respectively in WT and KO mice with ulcerative colitis [(C, n= 6 per group)].

**
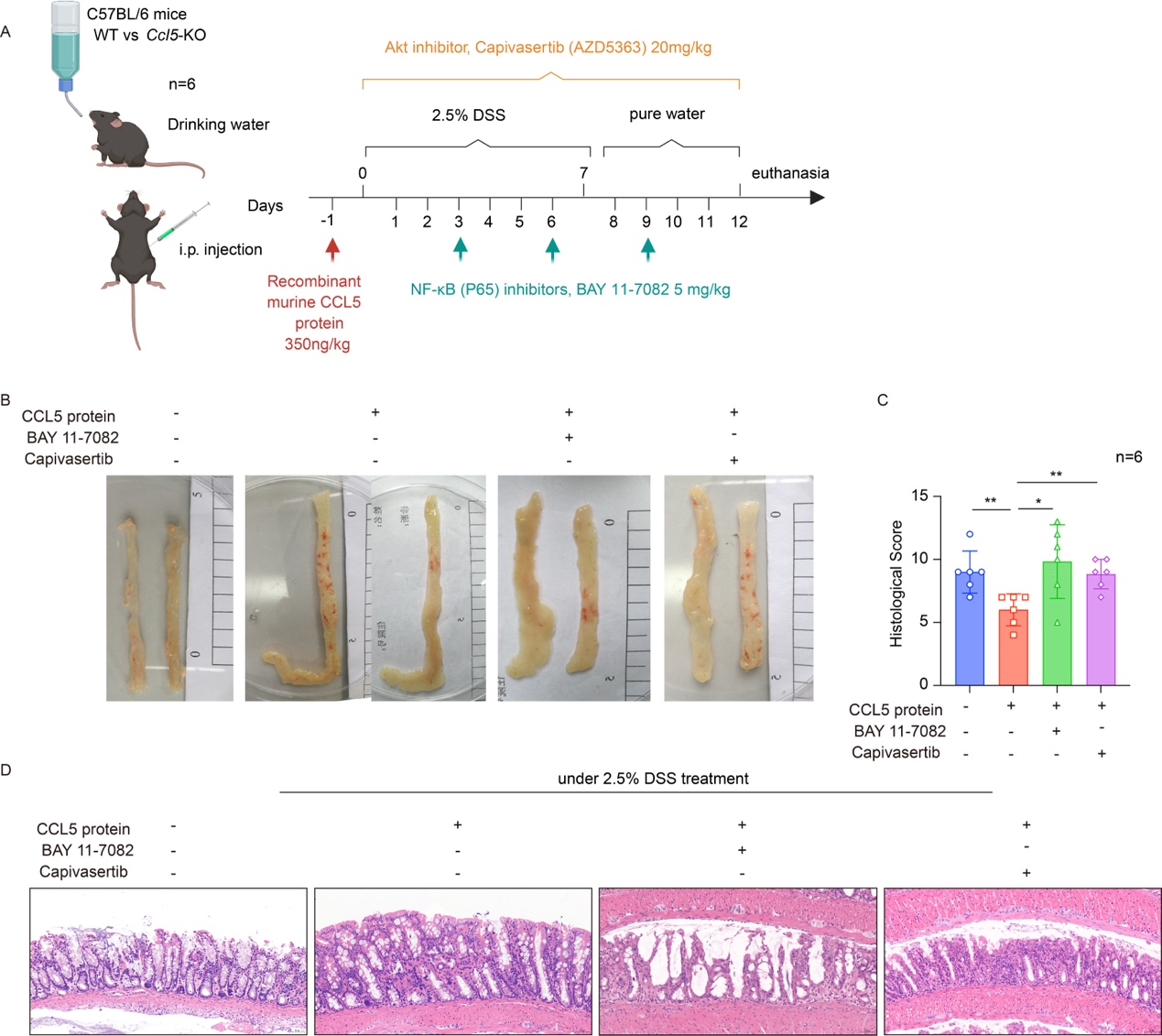
Supplementary Figure 3:**

(A) Schematic of *in-vivo* treatment of recombinant murine CCL5 protein (350ng/kg), NF-κB (P65) inhibitor, BAY 11-7082 (5mg/kg), and Akt inhibitor, Capivasertib (AZD5363) (20mg/kg) in WT and *Ccl5*-KO mice.

(B) Gross anatomy of colons in mice from different treated groups (control, CCL5 small protein, CCL5 small protein + BAY 11-7082, CCL5 small protein + Capivasertib).

(C and D) Representative H&E staining (D) of colon sections in mice from different treated groups; Scale bars, 100μm. Histological score (C) was quantified.

**
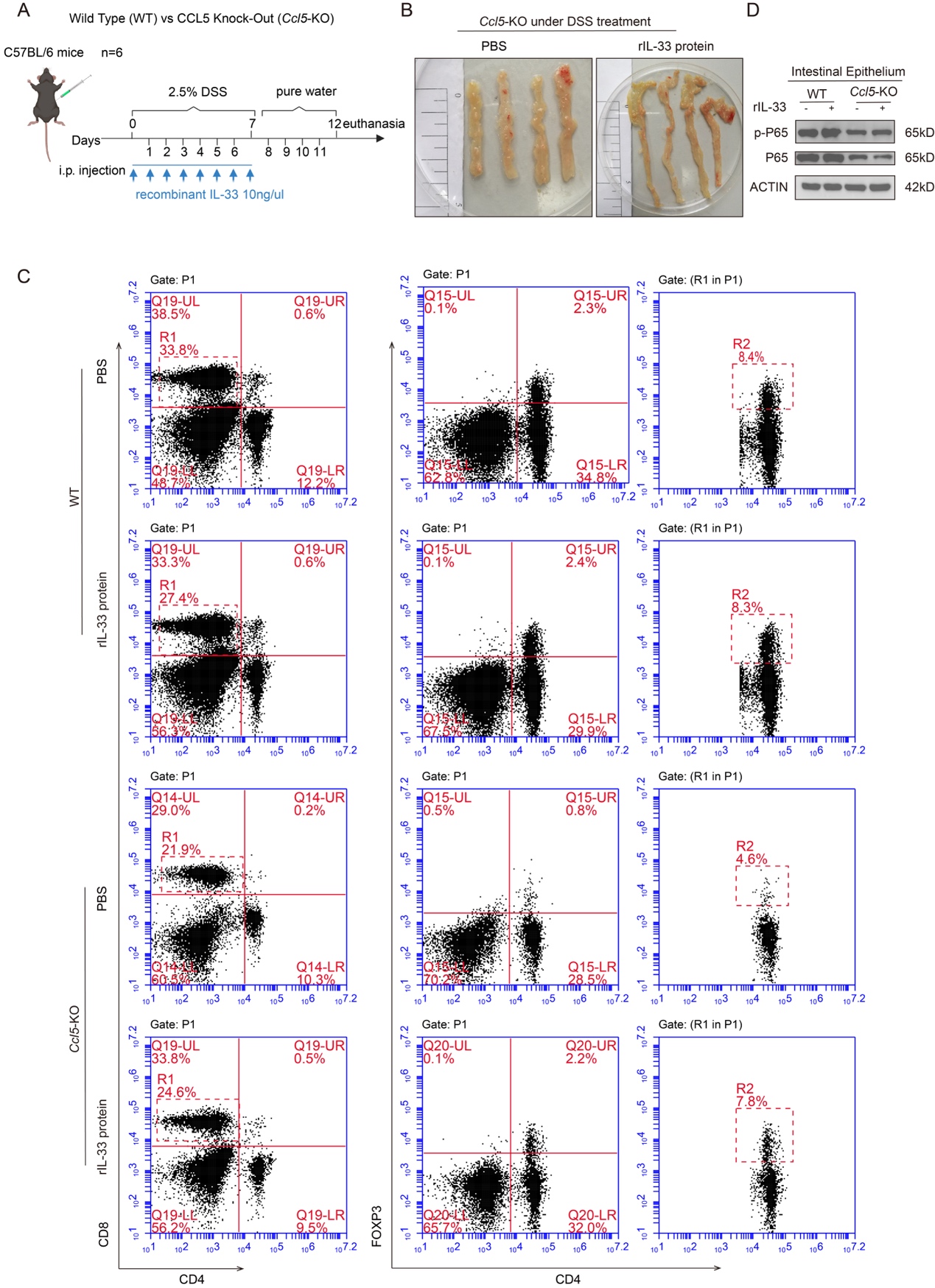
Supplementary Figure 4:**

(A) Schematic of *in-vivo* treatment of recombinant IL-33 (10ng/ul) in WT and *Ccl5*-KO mice.

(B) Gross anatomy of colons in mice received daily intraperitoneal injections of rIL-33 protein.

(C) Flow cytometry analysis of CD4^+^, CD8^+^, and FOXP3^+^ T cell numbers in the intestinal lymphoid tissues of *Ccl5*-KO and WT mice treated with PBS/rIL-33 after DSS treatment.

(D) Immunoblotting analysis of P65 and its phosphorylated protein levels in intestinal tissues of mice after PBS/rIL-33 treatment.


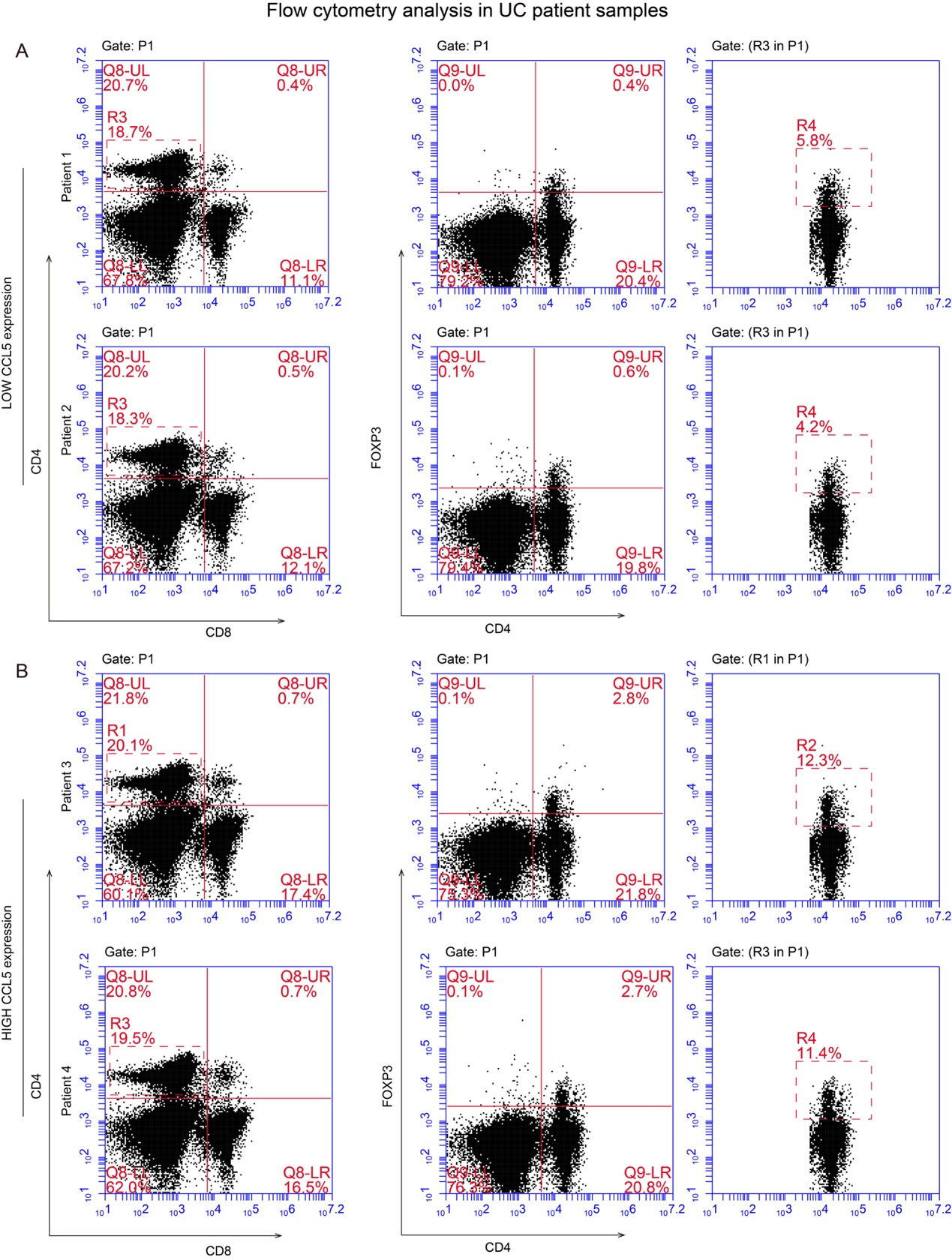
**Supplementary Figure 5:**

(A and B) Flow cytometry analysis of CD4^+^, CD8^+^, and FOXP3^+^ T cell numbers in inflammatory tissues of UC patients with high and low CCL5 levels.

**Supplementary table 1:** Patient Characteristic

|  | severe UC  (n=16 50%) | mild to moderate UC  (n=16 50%) | P value |
| --- | --- | --- | --- |
| Age, mean ± SD, yr | 35.63 ± 3.32 | 32.06 ± 2.58 | 0.404 |
| Sex |  |  |  |
| Male | 10 (62.50%) | 9 (56.25%) | 0.719 |
| Female | 6 (37.50%) | 7 (43.75%) |  |
| Extent of disease |  |  |  |
| Extensive colitis | 10 (62.50%) | 5 (33.33%) | 0.265 |
| Left side colitis | 4 (25.00%) | 7 (46.67%) |  |
| Proctitis | 2 (12.50%) | 3 (20.00%) |  |

**Supplementary table 2：**Histopathological scoring rubric used for colonic histopathological scoring.

| **Feature graded** | **Grade** | **Description** |
| --- | --- | --- |
| Inflammation | 0 | None |
|  | 1 | Slight |
|  | 2 | Moderate |
|  | 3 | Severe |
| Extent | 0 | None |
|  | 1 | Mucosa |
|  | 2 | Mucosa and submucosa |
|  | 3 | Transmural |
| Regeneration | 4 | No tissue repair |
|  | 3 | Surface epithelium not intact |
|  | 2 | Regeneration with crypt depletion |
|  | 1 | Almost complete regeneration |
|  | 0 | Complete regeneration or normal tissue |
| Crypt damage | 0 | None |
|  | 1 | Basal 1/3 damaged |
|  | 2 | Basal 2/3 damaged |
|  | 3 | Only surface epithelium intact |
|  | 4 | Entire crypt and epithelium lost |
| Percent involvement | 1 | 1-25% |
|  | 2 | 26-50% |
|  | 3 | 51-75% |
|  | 4 | 76-100% |
